# A New Phenolic Acid Decarboxylase from the Brown-Rot Fungus *Neolentinus lepideus* Natively Decarboxylates Biosourced Sinapic Acid into Canolol, a Bioactive Phenolic Compound

**DOI:** 10.3390/bioengineering11020181

**Published:** 2024-02-14

**Authors:** Elise Odinot, Alexandra Bisotto-Mignot, Toinou Frezouls, Bastien Bissaro, David Navarro, Eric Record, Frédéric Cadoret, Annick Doan, Didier Chevret, Frédéric Fine, Anne Lomascolo

**Affiliations:** 1 OléoInnov, 19 Rue du Musée, F-13001 Marseille, France; elise.odinot@oleoinnov.com; 2 INRAE, Aix-Marseille Université, UMR1163 BBF Fungal Biodiversity and Biotechnology, 163 Avenue de Luminy, F-13009 Marseille, France; alexandra.bisotto@gmail.com (A.B.-M.); bastien.bissaro@inrae.fr (B.B.); david.navarro@inrae.fr (D.N.); eric.record@inrae.fr (E.R.); cadfred86@gmail.com (F.C.); annick.doan@inrae.fr (A.D.); 3 INRAE, UMR1319 MICALIS Institute, PAPPSO, Domaine de Vilvert, F-78350 Jouy-en-Josas, France; didier.chevret@inrae.fr; 4 TERRES INOVIA, Parc Industriel, 11 Rue Monge, F-33600 Pessac, France; f.fine@terresinovia.fr

**Keywords:** biorefinery process, canolol, ferulic acid, *Neolentinus lepideus*, phenolic acid decarboxylase, rapeseed meal, sinapic acid, 4-vinylguaiacol

## Abstract

Rapeseed meal (RSM) is a cheap, abundant and renewable feedstock, whose biorefinery is a current challenge for the sustainability of the oilseed sector. RSM is rich in sinapic acid (SA), a *p*-hydroxycinnamic acid that can be decarboxylated into canolol (2,6-dimethoxy-4-vinylphenol), a valuable bioactive compound. Microbial phenolic acid decarboxylases (PADs), mainly described for the non-oxidative decarboxylation of ferulic and *p*-coumaric acids, remain very poorly documented to date, for SA decarboxylation. The species *Neolentinus lepideus* has previously been shown to biotransform SA into canolol in vivo, but the enzyme responsible for bioconversion of the acid has never been characterized. In this study, we purified and characterized a new PAD from the canolol-overproducing strain *N. lepideus* BRFM15. Proteomic analysis highlighted a sole PAD-type protein sequence in the intracellular proteome of the strain. The native enzyme (*Nle*PAD) displayed an unusual outstanding activity for decarboxylating SA (V_max_ of 600 U.mg^−1^, k_cat_ of 6.3 s^−1^ and k_cat_/K_M_ of 1.6 s^−1^.mM^−1^). We showed that *Nle*PAD (a homodimer of 2 × 22 kDa) is fully active in a pH range of 5.5–7.5 and a temperature range of 30–55 °C, with optima of pH 6–6.5 and 37–45 °C, and is highly stable at 4 °C and pH 6–8. Relative ratios of specific activities on ferulic, sinapic, *p*-coumaric and caffeic acids, respectively, were 100:24.9:13.4:3.9. The enzyme demonstrated in vitro effectiveness as a biocatalyst for the synthesis of canolol in aqueous medium from commercial SA, with a molar yield of 92%. Then, we developed processes to biotransform naturally-occurring SA from RSM into canolol by combining the complementary potentialities of an *Aspergillus niger* feruloyl esterase type-A, which is able to release free SA from the raw meal by hydrolyzing its conjugated forms, and *Nle*PAD, in aqueous medium and mild conditions. *Nle*PAD decarboxylation of biobased SA led to an overall yield of 1.6–3.8 mg canolol per gram of initial meal. Besides being the first characterization of a fungal PAD able to decarboxylate SA, this report shows that *Nle*PAD is very promising as new biotechnological tool to generate biobased vinylphenols of industrial interest (especially canolol) as valuable platform chemicals for health, nutrition, cosmetics and green chemistry.

## 1. Introduction

**Figure 1:**
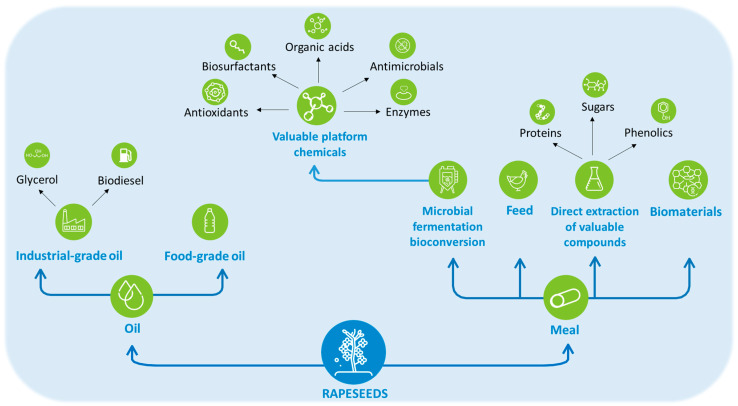
Schematic routes of rapeseed and RSM refinery.

Rapeseed (*Brassica napus*) is one of the world’s major oil crops after palm and soy. In 2023, the world crop culture area was estimated at 42 million hectares [1], with 92% of the seed production covered by the EU, Canada, China, India, Australia, Russia, Ukraine, USA and UK. In Europe, rapeseed oil is the main feedstock for biodiesel production. Rapeseed meal (RSM), the main solid residue of the rapeseed oil industry, is a natural, cheap (around USD 300–400 per ton) and abundant plant biomass with a world production estimated to 47 million tons in 2023 [1]. RSM contains high contents of total phenolic compounds (1–2% defatted dry matter, DDM) [2,3,4,5], which are mainly composed of sinapic acid esters, notably sinapine (sinapoyl choline, 80% of the total phenolic content). The other phenolics include mono-, di- and tri-sinapoyl esters of sugars and/or flavonoids such as kaempferol [6,7,8]. RSM is mainly composed of proteins (34–37%), fibers (lignocellulosic materials, 11.5–12.7%) and minerals (6.1–7%) [2,4]. RSM was first used as animal feed to complement monogastric and ruminant diets, but this use cannot absorb the huge yearly production of RSM. For two decades, research has focused on new routes of RSM valorization to produce valuable chemicals of industrial interest. As a result, RSM can now be integrated into a whole-crop refining scheme, including several valorization pathways: (i) direct extraction of proteins, sugars and phenolics; (ii) direct valorization as biomaterials and (iii) source of valuable platform molecules after microbial fermentation/bioconversion, such as antioxidants, antimicrobials, organic acids, biosurfactants and enzymes [2,4] (Figure 1). The biorefinery of oilseed meals is thus a crucial challenge to develop the sustainability of the agro-industrial sector.

**Figure 2:**
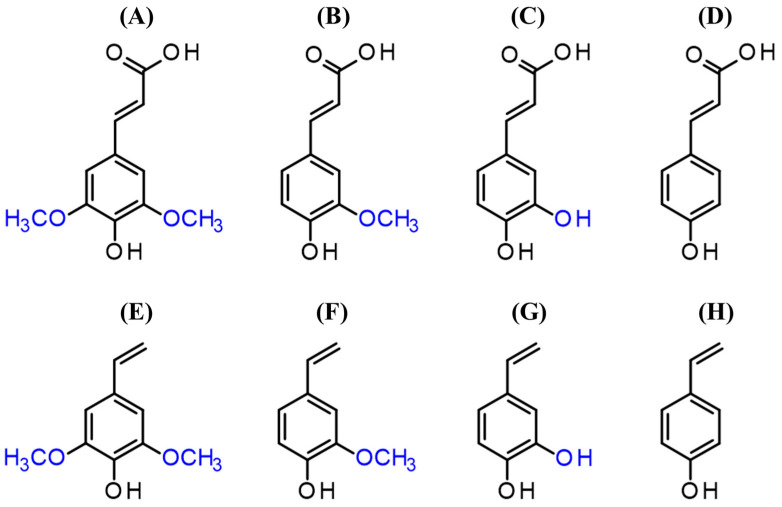
Chemical structure of *p*-hydroxycinnamic acids and their vinyl derivatives: (**A**) sinapic acid (3,5-dimethoxy-4-hydroxycinnamic acid), (**B**) ferulic acid (4-hydroxy-3-methoxycinnamic acid), (**C**) caffeic acid (3,4-dihydroxycinnamic acid), (**D**) *p*-coumaric acid (4-hydroxycinnamic acid), (**E**) canolol (2,6-dimethoxy-4-vinylphenol), (**F**) 4-vinylguaiacol (2-methoxy-4-vinylphenol), (**G**) 4-vinylcatechol (2-hydroxy-4-vinylphenol), (**H**) 4-vinylphenol.

Sinapic acid (SA, 4-hydroxy-3,5-dimethoxycinnamic acid) is the main phenolic acid that can be obtained from RSM (90–95% of total phenolics), and it belongs to the group of *p*-hydroxycinnamic acids (pHCAs). pHCAs, such as ferulic acid (FA, 4-hydroxy-3-methoxycinnamic acid), *p*-coumaric acid (pCA, 4-hydroxycinnamic acid), caffeic acid (CafA, 3,4-dihydroxycinnamic acid) and SA (Figure 2) can be commonly found in plant biomass and agro-residues including cereal brans and straws, sugar beet and coffee pulps, and oilseed meals [9]. Vinyl derivatives obtainable by decarboxylation of these pHCAs (Figure 2) could be suitable for an array of food, cosmetics or pharmaceutical applications due to their strong antioxidant and anti-inflammatory activities [10]. Canolol (2,6-dimethoxy-4-vinylphenol or vinylsyringol), the product of SA decarboxylation, was discovered and characterized about 20 years ago [11,12], as a natural phenolic compound occurring during the process of crude rapeseed oil extraction at high temperature, and preventing oil autooxidation. However, the canolol totally disappeared from the oil after the refining steps, which coincided with a decrease in the oil’s stability against autooxidation. Comparable or stronger antioxidant activities were found for canolol in comparison to other natural antioxidants such as α- and γ-tocopherol, ascorbic acid, β-carotene, rutoside and quercetin [11,12,13]. Moreover, due to its lipophilic nature, canolol is a bioactive antioxidant, soluble in fatty matrices. Canolol thus has potential applications in health, nutrition and cosmetics. For instance, canolol showed evidence of a preventive effect against various cancers [14]. Canolol could also be used as a precursor for thermoplastic biopolymers and natural biobased monomers for green chemistry (diepoxydized diphenyls) to advantageously serve as substitutes for the diglycidyl ether of bisphenol A [15,16]. As a result, the scientific and industrial community is showing increasing interest in canolol and its possible biosynthesis pathways from biobased SA.

Historically described in beer and whiskey [17], the microbial conversion of pHCAs into the corresponding vinylphenols occurs through the non-oxidative decarboxylation of pHCAs catalyzed by metal-independent cofactor-free intracellular enzymes named phenolic acid decarboxylases or PADs (for a review, see [9]). PADs (EC 4.1.1, carboxyl lyase family) would be involved in the microbial phenolic detoxification pathway. To date, only PADs from bacteria (*Bacillus, Lactobacillus*, *Pseudomonas* and *Enterobacter* genera) and certain yeasts (e.g., *Brettanomyces bruxellensis*, *B. anomalus*, *Candida guilliermondii*) have been thoroughly purified, and characterized as homodimeric enzymes of 40–46 kDa [18,19,20,21,22,23,24]. The crystalline structure of the PAD from *Bacillus pumilus* strain UI-670 (*Bpu*PAD) was solved as two monomers, each of them consisting of two α-helices and a β-barrel harboring within the active site, a hydrophobic cavity with highly conserved hydrophobic amino acids [25]. In *Lactobacillus plantarum*, it was shown that the PAD enzyme interacts with the pHCA substrate via notably the amino acids Glu71, Arg48, and two Tyr residues, Tyr18 and Tyr20 [26]. These amino acids are conserved hallmarks of the active site of bacterial and yeast PADs [26,27].

Decarboxylation of SA into canolol can be achieved via physico-chemical treatments of RSM, such as heat, pressure and alkaline treatments [28,29,30,31]. However, the conversion yields remain lower than 1 mg.g^−1^ meal, and the processes do not reliably scale up. Microbial PAD-mediated non-oxidative decarboxylation of SA could therefore be a ‘green’ alternative to produce canolol in suitable amounts for industrial applications. Bacterial and yeast PAD activity has essentially been described for the decarboxylation of FA and pCA as preferential substrates. Decarboxylation of the di-hydroxycinnamic acid CafA by PADs has also been reported [22,32], although with a much lower specific activity in general [9,24]. A PAD from *B. licheniformis* has also been shown to display an anecdotic activity on SA, with relative ratios of specific activities of 100:75.6:34.4:0.3 for pCA, FA, CafA and SA, respectively [24]. Site-directed mutagenesis of wild-type *B. pumilus* and *B. amyloliquefaciens* PADs (originally inactive on SA) made it possible to obtain evolved enzymes able to decarboxylate SA, but this activity remained very low compared to the activity on FA and pCA [33,34]. 

**Figure 3:**
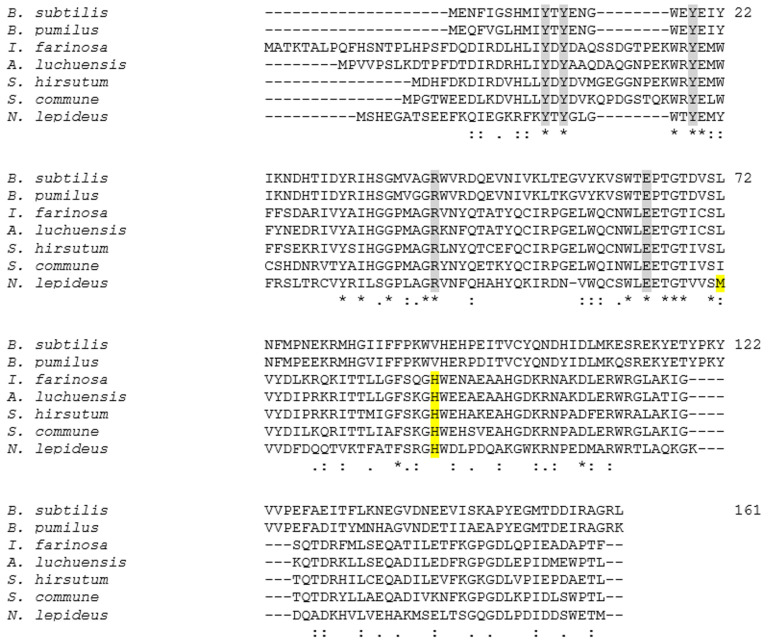
Comparison of bacterial and fungal phenolic acid decarboxylases (PADs). ClustalW alignment of the PAD protein sequences from *Bacillus subtilis*, *Bacillus pumilus*, *Aspergillus luchuensis*, *Isaria farinosa*, *Neolentinus lepideus*, *Schizophyllum commune* and *Stereum hirsutum* (Accession Numbers in the NCBI database: O07006.1, WP_099727689.1, CUI18215.1, BBC70792.1, KZT30061.1, QQD79822.1 and XP_007303961.1, respectively). The amino acids (aa) described as involved in the catalytic mechanism are indicated in grey boxed letters. These aa are highly conserved among the sequences. The yellow-colored aa correspond to the aa we hypothesized to be potentially determinant to explain the difference between specific activity of *Nle*PAD towards SA in comparison to the bacterial *Bsu*PAD. The numbering of aa is based on the sequence of the *B. subtilis* PAD [27]. The *N. lepideus* sequence is the protein sequence predicted from the publicly available genome of the strain *N. lepideus* HHB14362.

To date, the literature on PADs from filamentous fungi remains very scarce. To our knowledge, the endophytic fungus *Phomopsis liquidambari* was the first fungus to be described for transforming SA into canolol in vivo through PAD-type activity [35]. PADs have recently been characterized from the filamentous ascomycetes *Isaria farinosa* and *Aspergillus luchuensis,* and the basidiomycete *Schizophyllum commune* [36,37,38], but none of these enzymes showed activity on SA. In silico, some putative PAD sequences could be predicted in annotated publicly-available fungal genomes [39], particularly in the class Agaricomycetes, including the species *Neolentinus lepideus*, *Schizophyllum commune* and *Stereum hirsutum*. These sequences shared less than 50% similarity with those of bacterial and yeast PADs [9] (Figure 3). In 2017, we showed that the species *N. lepideus* was the only fungus able to biotransform, in vivo, all of the SA, FA, pCA and CafA supplemented in liquid culture media into the corresponding vinyl derivatives, namely canolol, 4-vinylguaicol (4-VG), 4-vinylphenol (4-VP) and 4-vinylcatechol [40,41], albeit with weaker activity on pCA and CafA. The strain *N. lepideus* BRFM15 was especially highlighted for the production of up to 1–1.5 g.L^−1^ canolol or 4-VG in submerged culture media fed daily with SA or FA, respectively, which suggested that this strain did possess a PAD with good affinity for these two pHCAs. We also showed that crude intracellular extracts of *N. lepideus* BRFM15 contained a PAD activity capable of decarboxylating both FA and SA at a temperature of 37 °C and a pH of 6.5 [40].

The aim of the work reported here was to purify and characterize the native intracellular PAD from *N. lepideus* strain BRFM15 (referred to hereafter as *Nle*PAD) and to estimate its biotechnological potential for the synthesis of canolol from biosourced SA extracted from RSM as a cheap, natural and abundant feedstock.

## 2. Materials and Methods

### 2.1. Chemicals

All chemicals, including 4-VG and 4-VP, were purchased from Sigma-Aldrich (Saint-Quentin Fallavier, France). Methyl sinapate was obtained from Apin Chemicals Ltd. (Compton, UK). Pure canolol was kindly provided by the Agropolymer Engineering and Emerging Technologies unit at the French National Research Institute for Agriculture, Food and Environment (INRAE IATE, Montpellier, France).

### 2.2. Microorganisms and Culture Conditions

The *Neolentinus lepideus* strain BRFM15 studied was deposited in the CIRM-CF collection (International Centre of Microbial Resources dedicated to Filamentous Fungi, INRAE, Marseille, France). It was kept on malt agar slants at 4 °C.

Precultures and cultures of *N. lepideus* BRFM15 were carried out as previously described by Odinot et al. [41]. In order to induce PAD activity, commercial SA was added to 3-day-old cultures as a filter-sterilized solution at a final concentration of 0.3 g.L^−1^, and was fed in daily to keep the final concentration in the culture medium at 0.3 g.L^−1^. 

The recombinant strain *Aspergillus niger* BRFM451 is a feruloyl esterase A (*An*FaeA) overproducing strain, formerly engineered in our laboratory from the host strain *A. niger* D15#26 [42]. In this study, this strain was used to produce batches of *An*FaeA enzyme for use in bioconversion trials on biosourced SA from RSM. The culture medium was buffered at pH 5 with a 0.1 M citrate-sodium phosphate buffer, and the production of the recombinant *An*FaeA enzyme was triggered by a concentration of 50 g.L^−1^ glucose [42].

### 2.3. N. lepideus BRFM15 Intracellular Proteome Analysis

After a 10-day cultivation of *N. lepideus* BRFM15 grown in the presence of SA as PAD inducer, the mycelium was separated from the culture broth by filtration on 0.22-µm glass-fiber filters, rinsed with deionized water and ground with liquid nitrogen. One hundred milligrams of the resulting powder was dissolved in a Tris-HCl 100 mM pH 7.4 lysis buffer containing 4% (*w*/*v*) sodium dodecyl sulfate, 2% (*w*/*v*) dithiothreitol (DTT), 20% (*v*/*v*) glycerol and 20 mM phenylmethanesulfonyl fluoride (PMSF), then incubated at 95 °C for 15 min. After centrifugation at 11,300× *g* for 10 min, the resulting supernatant was mixed with trichloroacetic acid (TCA) 10% (final volume) to precipitate proteins. The proteins were then separated by 1D electrophoresis per the protocol of Couturier et al. [43]. After protein lysis with trypsin, peptide analysis was performed by liquid chromatography–tandem mass spectrometry (LC-MS/MS) at the PAPPSO platform facility (INRAE, Jouy-en-Josas, France) as described by Arfi et al. [44]. Based on the list of peptides, protein identification was performed by querying the MS/MS data against the predicted proteins obtained from publicly-available *N. lepideus* HHB14362 genome data [45]. The genome of strain *N. lepideus* HHB14362 was sequenced and annotated in 2016 by the US Department of Energy Joint Genome Institute (JGI) [46]. NCBI Accession Number for the predicted protein sequence of the PAD from *N. lepideus* HHB14362 was KZT30061.1, corresponding to JGI accession number 1126845 [45], further supported by the corresponding predicted PAD gene (536 bp) and cDNA (489 bp) sequences (Additional File 1: Figure S1).

### 2.4. NlePAD Purification

Mycelium (34.0 g wet mass) from a 2.6-L culture of *N. lepideus* BRFM15, grown as described above in the presence of SA as PAD inducer, was collected by filtration through GF/F glass-fiber filters (Whatman, Maidstone, UK). The mycelium was then resuspended in 130 mL of sodium phosphate buffer (20 mM, pH 7.5) containing 0.4 M saccharose and 2 mM DTT (buffer A) and mixed with 95 g Fontainebleau sand (Sigma). Cells were then broken with an Ultra-Turrax blender (13,500 rpm, 3 min) on ice, and cell debris was removed by two successive centrifugations at 10,000× *g* for 30 min followed by filtration through GF/D glass-fiber filters. The resulting supernatant constituted the cell-free extract and was immediately used for enzymatic activity assay and purification, or kept at −20 °C with 20% (*v*/*v*) glycerol.

The crude cell-free extract was poured into a DEAE-Sepharose Fast Flow chromatography column (gel volume 60 mL, 2.6 × 11.3 cm, GE Healthcare Bio-Sciences AB, Uppsala, Sweden), pre-equilibrated with buffer A at a flow rate of 1 mL.min^−1^. The column was washed with 167 mL of buffer A, and the unbound proteins (containing PAD) were recovered and concentrated using a 10 kDa polyethersulfone (PES) membrane. The concentrated solution was then loaded on a Sephacryl S-100HR column (2.6 cm × 92 cm; GE Healthcare) pre-equilibrated with buffer A at a flow rate of 0.5 mL.min^−1^. Proteins were eluted with buffer A at a flow rate of 0.5 mL.min^−1^ in 3.5-mL fractions. Active fractions were pooled and concentrated using a 10-kDa PES membrane. The solution was then loaded onto a Superdex 75 Prep Grade column (1.6 × 60 cm; GE Healthcare) pre-equilibrated with buffer A at a flow rate of 0.5 mL.min^−1^. Proteins were eluted with buffer A at a flow rate of 0.5 mL.min^−1^ in 1-mL fractions. The active fractions were pooled, concentrated using a 10-kDa PES membrane, and stored at −20 °C with 20% (*v*/*v*) glycerol.

To determine the molecular mass of the native *Nle*PAD, the purified enzyme was applied to the Superdex 75 Prep Grade gel filtration column (same as above) equilibrated with buffer A. Calibration used a solution of molecular standards (10 mg.mL^−1^ of each protein): bovine serum albumin (66 kDa), α-amylase (53 kDa), ovalbumin (43 kDa), casein (27 kDa) and lysozyme (13.5 kDa). The K_av_ for PAD was determined as the ratio V_e_ − V_0_/V_t_ − V_0_, where V_e_ is elution volume measured for PAD, V_0_ is column void volume (39.81 mL), and V_t_ is total column volume (120.64 mL).

Protein concentration was determined according to Bradford [47], with bovine serum albumin (BSA) as the standard. SDS-PAGE was performed on 12% acrylamide gels in order to control-check the efficiency of the purification steps. Proteins were detected by a standard silver staining method, and the PageRuler™ Prestained Protein Ladder (ThermoFisher Scientific, Illkirch, France) was used as the molecular mass standard.

### 2.5. Assay for PAD Activity

*Nle*PAD activity was determined in a reaction mixture of 100 µL enzyme (crude preparation or purified enzyme, corresponding to 0.5–1 µg enzyme) and 100 µL sodium phosphate buffer (100 mM, pH 6) containing 4 mM substrate (SA or FA). Incubation was carried out for 30 min at 37 °C for SA as substrate or 45 °C for FA as substrate (standard reaction). The reaction was stopped by adding 12.2 µL acetic acid and 100 µL methanol. Quantification of the enzymatic product (canolol or VG) was performed by HPLC analysis as described below. Enzyme activity was expressed in Units (U), where 1 U was defined as the quantity of enzyme that produced 1 µmol canolol (or VG) per hour.

### 2.6. Assay for AnFaeA Activity

*An*FaeA activity was assayed spectrophotometrically at 37 °C, as previously described [48], by monitoring A_335_ with respect to the rate of hydrolysis of 0.032 mM of the enzyme substrate in 100 mM of sodium phosphate buffer (pH 6). The model substrate used was methyl sinapate. The extinction coefficients at 335 nm were 13,318 L.mol^−1^.cm^−1^ for methyl sinapate and 5500 L.mol^−1^.cm^−1^ for SA [48]. Enzyme activity was expressed in nanokatals (1 nkat corresponds to the amount of enzyme able to hydrolyze 1 nmol of substrate per second). The experiments were performed in triplicate, and the standard deviation was lower than 5% of the mean.

### 2.7. Effect of pH, Temperature and Organic Solvents on NlePAD Activity and Stability

To determine the pH optimum, *Nle*PAD activity was assayed as in the standard reaction but with pH values made to range from 5.5 to 7.5 (sodium phosphate buffers). To determine the temperature optimum, the standard activity assay was performed at temperatures from 30 °C to 60 °C. 

The effect of temperature on enzyme stability was studied by incubating purified *Nle*PAD for 1 h to 120 h at temperatures of 4, 30, 37, 45 and 55 °C. The effect of pH on enzyme stability was studied by incubating purified *Nle*PAD for 3 h to 10 days at pH 4 to 8. After these treatments, residual enzyme activity was determined under standard conditions. The effect of organic solvents (ethanol, methanol, acetonitrile) was determined using standard assay conditions in the presence of 0–40% (*v*/*v*) ethanol, methanol, or acetonitrile. 

### 2.8. In Vitro Bioconversion of Commercial SA into Canolol

In vitro bioconversion assays were carried out in duplicate in 2-mL screw-capped tubes at 37 °C for 24 h. The reaction mixture was composed of 175 µL *Nle*PAD (0.14 or 0.30 U) and 175 µL sodium phosphate buffer (100 mM, pH 6) containing various concentrations (1.4 to 3.2 mM) of commercial SA. At 0.5, 4, 6, 8 and 24 h of incubation, a 50 µL aliquot was removed from the reaction medium, and the reaction was stopped with 4 µL of acetic acid and 50 µL of methanol. The concentrations of SA and canolol in these samples were then analyzed by HPLC as described below.

### 2.9. In Vitro Bioconversion of Biosourced SA from RSM into Canolol

The RSM used here as natural source of biosourced SA was provided by the Technical Centre for Oilseed Crops, Grain Legumes and Industrial Hemp (TERRES INOVIA, Pessac, France). This RSM was obtained by seed pressing and hexane solvent oil extraction. Further industrial processing steps included: preconditioning at about 45 °C, heating at 95–100 °C for 60 min, then steam desolventizing at 107 ± 2 °C for 80 ± 5 min [49]. Traces of residual oil (around 1–2% dry matter) were then eliminated in our laboratory by hexane extraction with stirring for 48 h, filtration and hexane evaporation for 48 h.

**Figure 4:**
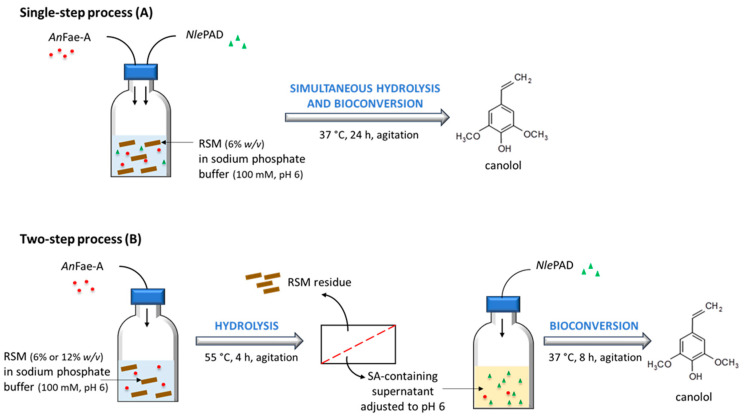
Schematic routes of the in vitro bioconversion of biosourced SA from RSM into canolol.

In a first set of experiments, the bioconversion of biosourced SA from RSM into canolol was carried out using a single-step process (Figure 4A). Sixty milligrams of RSM (i.e., 6% *w*/*v*) was incubated in 1 mL 100 mM sodium phosphate buffer (pH 6) supplemented with 39 nkat *An*FaeA (adapted from [41]) per gram of RSM plus 1.714 U purified *Nle*PAD (as measured on SA). The reaction mixture was incubated at 37 °C under agitation for 24 h. After 0.5, 1, 3.5, 6, 8 and 24 h of incubation, a 100-µL aliquot was removed from the reaction medium, and the reaction was stopped with 20 µL of acetic acid and 125 µL of methanol. The corresponding reaction mixture was then filtered (Restek^®^ polyvinyldifluoride syringe 0.45 µm-filters, RestekFrance, Lisses, France) to remove meal residues. A control was carried out using the same reaction medium but with *An*FaeA only in order to control-check the hydrolyzing activity of the enzyme on RSM (control 1). Another control (control 2) was performed using the same reaction medium but with *Nle*PAD only. In all cases, the SA and canolol concentrations in the samples were analyzed by HPLC as described below.

In a second set of experiments, the bioconversion of biosourced SA from RSM into canolol was performed in two successive steps (Figure 4B). In the first step, RSM (6% or 12% *w*/*v*) was incubated in 200 mL sodium phosphate buffer (100 mM, pH 6) supplemented with 39 nkat *An*FaeA per gram of RSM. The mixture was incubated at 55 °C for 4 h under agitation to release free SA. The reaction mixture was then filtered on Millipore Calbiochem^®^ Miracloth paper (Merck France, Saint-Quentin-Fallavier, France) and centrifuged at 7000× *g* for 20 min. The pH of the resulting supernatant, containing free SA, was then adjusted to 6 with NaOH. In the second step, 200 µL of the purified *Nle*PAD (0.343 U PAD as measured on SA) was added to 200 µL of the SA-containing supernatant, and the mixture was incubated at 37 °C under agitation for 24 h. After 0.5, 2, 4, 6, 8 and 24 h of incubation, a 50-µL aliquot was removed from the reaction medium, and the reaction was stopped with 5 µL of acetic acid and 50 µL of methanol. For the second step of the process, we performed two separate controls, where the PAD substrate used was either commercial SA or natural SA isolated and purified from RSM (after *An*FaeA hydrolysis) and dried to powder. The first control consisted of incubating 200 µL of 1.381 mM or 2.430 mM commercial SA solution (100 mM sodium phosphate buffer, pH 6) and 200 µL of purified *Nle*PAD at 37 °C under agitation for 24 h. The second control consisted of replacing commercial SA by purified biosourced SA previously extracted from enzymatically-hydrolyzed RSM, concentrated and dried to powder, and incubating in the same conditions as in the first control. In all cases, SA and canolol concentrations in the samples were analyzed by HPLC as described below.

### 2.10. High Performance Liquid Chromatography (HPLC) Analysis of the Monomeric Phenolics from NlePAD Reaction Medium

HPLC analysis of monomeric phenolic compounds was performed at 220 nm and 30 °C on a model Agilent 1100-series HPLC system (Agilent Technologies, Massy, France) equipped with a variable UV/Vis detector and 100-position autosampler/autoinjector sampling (5 µL injection) as in Odinot et al. [41]. Separation was achieved on a C30 reversed-phase column (YMC™ Carotenoid 3 µm, 4.6 × 150 mm; Waters, Guyancourt, France). The mobile phases used (flow rate of 0.8 mL.min^−1^) were solvent A: water acidified with 0.05% phosphoric acid and acetonitrile (95:5, *v*/*v*), and solvent B: acetonitrile 100%. The gradient elution program was as follows: 10% B for 4 min, 10% B to 40% B (9 min), 40% B to 100% B (1 min), 100% B (4 min). Total run time was 18 min. The Agilent 1100-series ChemStation processed the data, and the quantification was performed by external standard calibrations.

### 2.11. Bioinformatic Analysis

Multiple sequence alignment was done using the ClustalW prediction software [50]. Structural homology model of *Nle*PAD was generated with AlphaFold2 v2 [51]. Patches of charge (calculated at pH 6.3) and hydrophobicity across the protein surface were computed with the “protein-sol patches” online software [52]. All structures were visualized with the PyMOL v2.5 software [53]. 

## 3. Results

### 3.1. Detection and Identification of the PAD from N. lepideus BRFM15 after Proteomic Analysis

**Figure 5:**
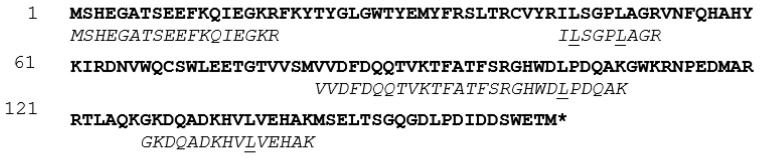
Comparison between PAD protein sequences from *N. lepideus* HHB14362 and *N. lepideus* BRFM15. ClustalW alignment of the protein sequence of the *N. lepideus* HHB14362 PAD (in bold), predicted from the publicly-available genome for this strain, and the peptide sequences from the PAD of *N. lepideus* BRFM15 identified by LC-MS/MS (in italics) after proteomics analysis. The underlined amino acids are either a leucine or an isoleucine, as the LC-MS/MS method used here was unable to firmly differentiate the two amino acids.

The intracellular proteome of *N. lepideus* BRF15, grown in the presence of SA as a PAD inducer in liquid cultures, was extracted from 10-day-old mycelium and analyzed by LC-MS/MS. Overall, about 850 proteins could be detected (see Additional File 2: Table S1) and identified by mass-matching against a database derived from the publicly-available genome annotation of the *N. lepideus* strain HHB14362 [46]. This genome contains a single predicted PAD (NCBI accession number KZT30061). In the proteome of the *N. lepideus* BRFM15 strain, a single protein corresponding to a PAD could be detected (see Additional File 2: Table S1). Interestingly, according to the semi-quantitative proteomic analysis based on spectra numbers, the abundance of this protein was 4-fold higher in the proteome from the PAD-inducing condition compared to the reference culture (i.e., without any inducer) (see Additional File 2: Table S1). The N-terminal amino acid sequence of the *Nle*PAD, together with four internal peptide sequences, were determined as MSHEGATSEEFKQIEGKR, IISGPIAGR, VVDFDQQTVKTFATFSR, GHWDIPDQAK and GKDQADKHVIVEHAK, respectively, and represented about 42% of the total protein, by comparison with the total amino sequence length of the predicted PAD from the genome of *N. lepideus* HHB14362 (Figure 5). Not considering the I and L amino acids (which were not distinguishable with the used LC-MS/MS method), sequence analysis of the detected peptides from *N. lepideus* BRFM15 PAD showed 100% sequence identity with the corresponding predicted peptides from the *N. lepideus* HHB14362 PAD. Of note, the N-terminal amino acid sequence from the PAD of *N. lepideus* BRFM15 showed 5.5% to 28% similarity with those of known yeast and bacterial PADs.

### 3.2. Purification of NlePAD

**Figure 6:**
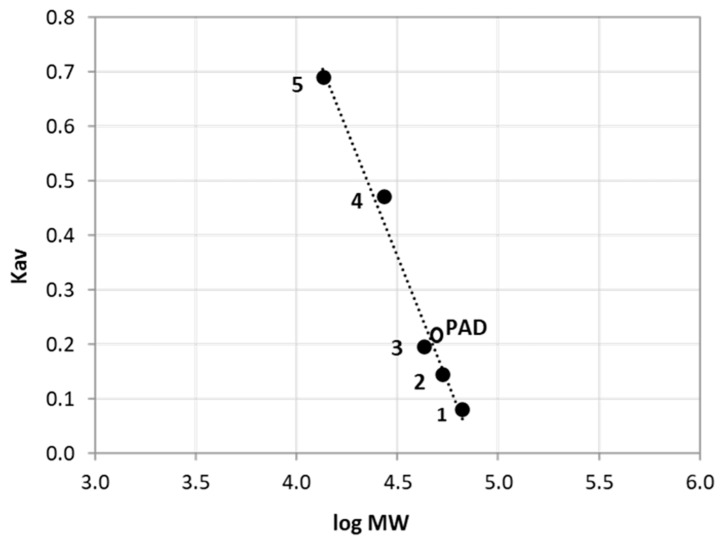
Molecular mass of the native *Nle*PAD. SEC was performed on Superdex S75 Prep Grade column. Protein markers are indicated as follows: (1) bovine serum albumin 66 kDa, (2) α-amylase 53 kDa, (3) ovalbumin 43 kDa, (4) casein 27 kDa and (5) lysozyme 13.5 kDa.

The *Nle*PAD was purified according to the procedure summarized in Table 1. The crude enzyme preparation was obtained after grinding mycelium and isolating the intracellular fluid. The enzyme was further purified by anion-exchange chromatography and two steps of size-exclusion chromatography (SEC). Following DEAE-Sepharose chromatography of the crude extract, PAD activity was recovered in the unbound proteins with a roughly three-fold increase in specific activity. It is worth noting that *Nle*PAD could not bind to any of the anion- or cation-exchange resins (e.g., DEAE-Sepharose, Q-Sepharose, carboxymethylcellulose) tested in our conditions. One hypothesis might be that the surface charges of *Nle*PAD seemed overall low and relatively heterogeneously distributed (predicted charges in Figure S6). After SEC using a Sephacryl S-100HR column, PAD activity was recovered in a single peak in fractions 50–72, corresponding to elution volumes ranging from 224 to 301 mL (Additional File 1: Figure S2A). The most active fractions (55–63, corresponding to elution volumes between 241.5 and 269.5 mL) were pooled. Analysis of this pool by SDS-PAGE showed a band of about 22 kDa (Additional File 1: Figure S2C, lane 3), which was close to the theoretical molecular mass of the predicted PAD from *N. lepideus* HHB14362 (18.916 kDa). This pool still contained some contaminants (Additional File 1: Figure S2C, lane 3), but its specific activity was increased 20-fold (Table 1). We therefore proceeded to a third purification step using a Superdex 75 Prep Grade column. PAD activity eluted as a single peak in fractions 46–66 corresponding to elution volumes of 46 to 66 mL (Additional File 1: Figure S2B). The most active fractions (57–60, corresponding to elution volumes between 57 and 60 mL) were pooled. Although this pool still contained a few contaminants (main one at about 30 kDa; Additional File 1: Figure S2C, lane 4), the three purification steps enabled us to isolate a purified PAD (band of 22 kDa) with a final yield of 22% and a 79-fold increase in specific activity (Table 1). The first step of SEC reduced the contaminant protein content by about 95% while the following SEC eliminated about 67% of the remaining proteins (Additional File 1: Figure S2A,B), giving a roughly estimated degree of purity of 98% after all the purification steps. This purified *Nle*PAD was used for further characterization. SEC analysis on a Superdex 75 Prep Grade column allowed us to determine the molecular mass of the native protein as 43–45 kDa (Figure 6), which suggests that the enzyme was a homodimeric protein of ~2 × 22 kDa. 

### 3.3. NlePAD Characterization

The purified enzyme was shown to be active on all four *p*-hydroxycinnamic acids tested, i.e., SA, FA, CafA and pCA, with relative ratios of specific activities (measured at 37 °C and pH 6) on FA, SA, pCA and CafA of 100:24.9:13.4:3.9, respectively. In this case, enzymatic activity was evaluated by measuring the disappearance of the substrate over time. Indeed, it has not been possible to obtain a commercial standard for 4-vinylcatechol, the decarboxylation product of CafA, but the *Nle*PAD production of 4-vinylcatechol was confirmed by LC-MS (Additional File 1: Figure S3). Canolol, 4-VG and 4-VP, detected in the *Nle*PAD-catalyzed bioconversion mixtures from SA, FA and pCA, respectively, were confirmed here by UV-Vis spectra and comparison against standards (Additional File 1: Figure S4). 

**Figure 7:**
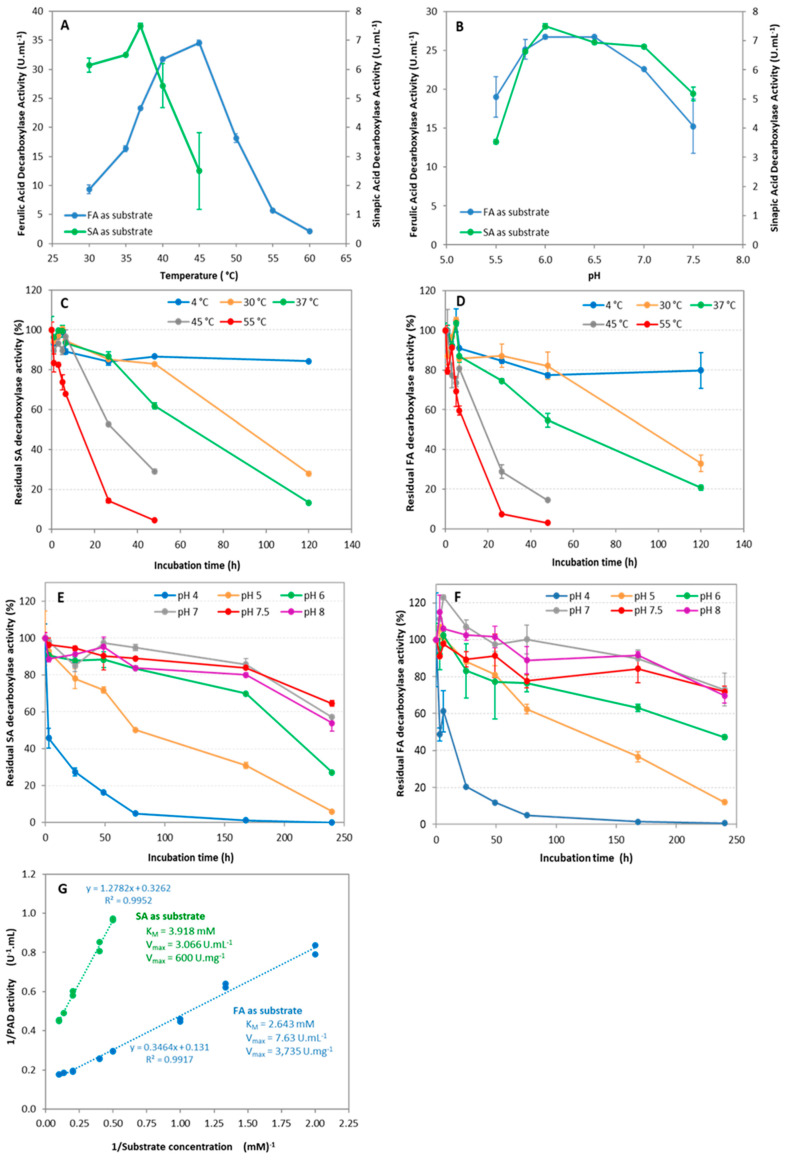
Characterization of *Nle*PAD. Effect of temperature (**A**) and pH (**B**) on the activity of *Nle*PAD, for SA and FA decarboxylation. Standard reaction conditions: 50 mM sodium phosphate buffer (pH 6), 2 mM substrate (SA or FA), 30 min incubation. Influence of temperature on the stability of *Nle*PAD, for the decarboxylation of SA (**C**) or FA (**D**)**.** One hundred percent of activity refers to 2.46 (±0.12) U.mL^−1^ (**C**) and 12.19 (±0.14) U.mL^−1^ (**D**), in standard conditions. Influence of pH on the stability of *Nle*PAD, for the decarboxylation of SA (**E**) or FA (**F**). One hundred percent activity refers to 1.22 (±0.02) U.mL^-1^ (**E**) and 9.88 (±0.86) U.mL^−1^ (**F**) in standard conditions. Influence of substrate concentration on the activity of *Nle*PAD, for SA and FA decarboxylation; Lineweaver-Burk plot (**G**). Standard reaction conditions: 50 mM sodium phosphate buffer (pH 6), substrate (SA or FA) ranging from 0.5 to 10 mM, 30 min incubation at 37 °C (SA) or 45 °C (FA). The enzyme concentration was 2 and 5 µg.L^−1^ for FA and SA assays, respectively. All assays were performed in duplicate.

The characteristics of the purified enzyme were systematically determined with both SA and FA as substrates for activity (Table 2). Under the conditions tested, the *Nle*PAD was active in the temperature range of 30–55 °C with an optimum at 37 °C for the decarboxylation of SA into canolol and 45 °C for the decarboxylation of FA into 4-VG (Figure 7A). The *Nle*PAD was shown to be almost fully active in a pH range of 5.5–7.5, with optimal activity at pH 6–6.5 with FA as substrate and at pH 6–7 with SA as substrate (Figure 7B). The *Nle*PAD was shown to be stable below 37 °C for several hours (Figure 7C,D). The enzyme was also shown to be stable for several days at 4 °C. Its half-life was about 90, 60, 23 and 11 h on average at 30 °C, 37 °C, 45 °C and 55 °C, respectively. Moreover, it was highly stable at pH values between 6 and 8, retaining 80–100% activity after incubation for 2 to 7 days at these pH values (Table 2, Figure 7E,F). By varying the concentration of SA or FA in the reaction mixture at pH 6, the apparent Michaelis constant (K_M_) and the maximum reaction velocity (V_max_) were determined (Figure 7G). K_M_ values were similar for both SA and FA (Table 2), which indicated that the enzyme shared the same affinity for both substrates, while the V_max_ values were 600 and 3735 U.mg^−1^, respectively. These values corresponded to a catalytic constant k_cat_ that was 6.2-fold higher for the conversion of FA into 4-VG (39.2 s^−1^) than for the conversion of SA into canolol (6.3 s^−1^), which indicated that the duration of the PAD catalytic cycle was about 6 times shorter for FA than for SA. The kinetic efficiency (or k_cat_/K_M_) was about 9-fold higher for FA than for SA.

**Figure 8:**
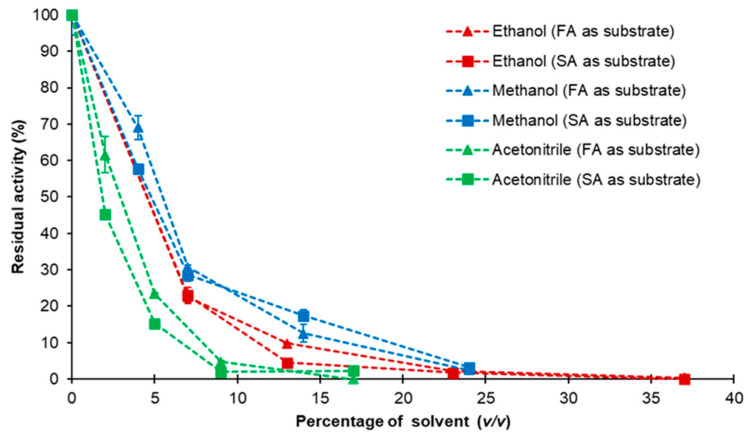
Influence of solvents on *Nle*PAD activity. Residual *Nle*PAD activity was determined in the presence of increasing concentrations (*v*/*v*) of ethanol, methanol or acetonitrile. The in vitro effect of the solvents was studied by incubating purified *Nle*PAD with each solvent at concentrations ranging from 0 to 40% (*v*/*v*) in the reaction mixture, using SA or FA as substrate, under standard conditions.

The organic solvent tolerance of *Nle*PAD towards ethanol, methanol and acetonitrile was studied using SA or FA as substrate (Figure 8). Ethanol, methanol and acetonitrile are water-miscible solvents, capable of solubilizing *p*-hydroxycinnamic acids and/or their corresponding vinylphenols (e.g., SA and canolol), and they are compatible with the conditions of our HPLC analyses. Consequently, they were chosen to be tested with the intention of carrying out further tests of inhibition of *Nle*PAD by the substrate or the product of the reaction. Ethanol, methanol and acetonitrile generally had a strong inhibitory effect on *Nle*PAD activity, even at low concentrations. Residual activity was lower than 10% and 20% for concentrations of about 15% (*v*/*v*) ethanol and methanol, respectively, and lower than 5% for a concentration of only 9% (*v*/*v*) acetonitrile. Solvent concentrations higher than 20–25% (*v*/*v*) of methanol or ethanol and higher than 10% of acetonitrile totally inhibited *Nle*PAD activity. 

### 3.4. Comparison of the Predicted Structure of NlePAD with Bacterial PADs Shows Differences in Active Site

**Figure 9:**
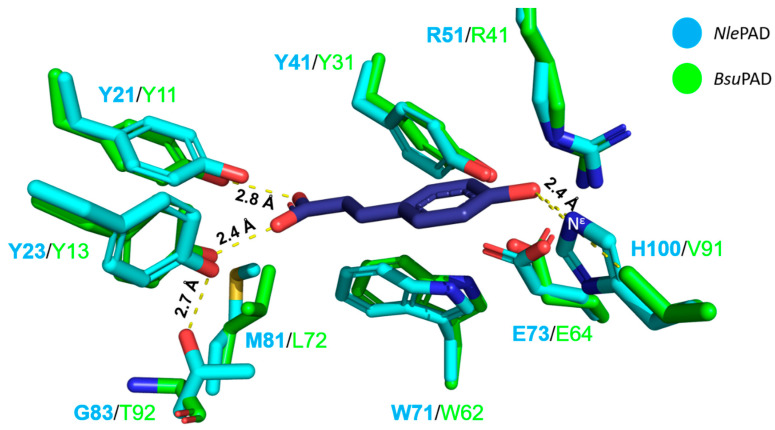
Superimposition of *Nle*PAD and *Bsu*PAD-Y19A mutant in complex with pCA. The figure shows the active site of *Nle*PAD (shown as blue cartoon; AlphaFold2 model, ref. [51]) and *Bsu*PAD (shown as green cartoon; PDB code 4ALB, ref. [27]) where pCA is shown as a dark purple stick. The catalytic residues are R51/E73 and R41/E64, and the substrate-binding tyrosines are Y21/Y23 and Y11/Y13 in *Nle*PAD and *Bsu*PAD, respectively. Note that for the sake of clarity, the amino acid main chains are hidden, except for G83.

To further understand the molecular determinants underlying the unprecedented activity of *Nle*PAD on SA, we carried out a comparative analysis of the predicted structure of the PAD from the *N. lepideus* strain HHB14362 (AlphaFold2 model, [51]; Additional File 1: Figure S5) with the crystallographic structures of the characterized PADs from *Bacillus pumilus* (*Bpu*PAD; PDB code 3NAD; [25]) and *Bacillus subtilis* (*Bsu*PAD; PDB code 4ALB; [27]). Note that the genome of strain BRFM15 is not sequenced yet, but peptides of PAD from both strains BRFM15 and HHB14362, detected by proteomics, showed 100% identity. While the overall fold is very similar (Additional File 1: Figure S6), one can observe some variations in the predicted charge distribution at the surface (Additional File 1: Figure S6A), and, more strikingly, a slightly more hydrophobic active site entrance (Additional File 1: Figures S6B and S7). This feature seems in line with the more non-polar nature of SA compared to pCA. On closer examination of the active site cavity, *Nle*PAD showed major differences from its orthologs (Figure 9 and Additional File 1: Figure S8). First, the neighborhood of the two tyrosines that establish hydrogen bonds with the carboxylic acid moiety of the substrate is different (Additional File 1: Figure S8A,B). Notably, the hydroxyl function of the side chain of Y23 (in *Nle*PAD) is in close vicinity of several residues that are drastically different in *Bsu*PAD (L72 and T92 in *Bsu*PAD are replaced by M81 and G83 in *Nle*PAD, respectively). Regarding neighbors of the catalytic Arg/Glu residues (Figure 9 and Additional File 1: Figure S8C,D), one remarkable natural mutation is the replacement of V91 in *Bsu*PAD by the bulkier H100 in *Nle*PAD. It is very likely that this substitution alters the substrate recognition/catalysis, as superimposition of the *Bsu*PAD-pCA complex and *Nle*PAD structures suggests that the H100 side chain would interact with the hydroxyl function of the substrate (His100-N^ε^–O-H (pCA) distance of 2.5 Å). Altogether, these natural mutations may contribute to *Nle*PAD activity on SA.

### 3.5. PAD-Catalyzed Bioconversion of Commercial and Biosourced SA into Canolol 

**Figure 10:**
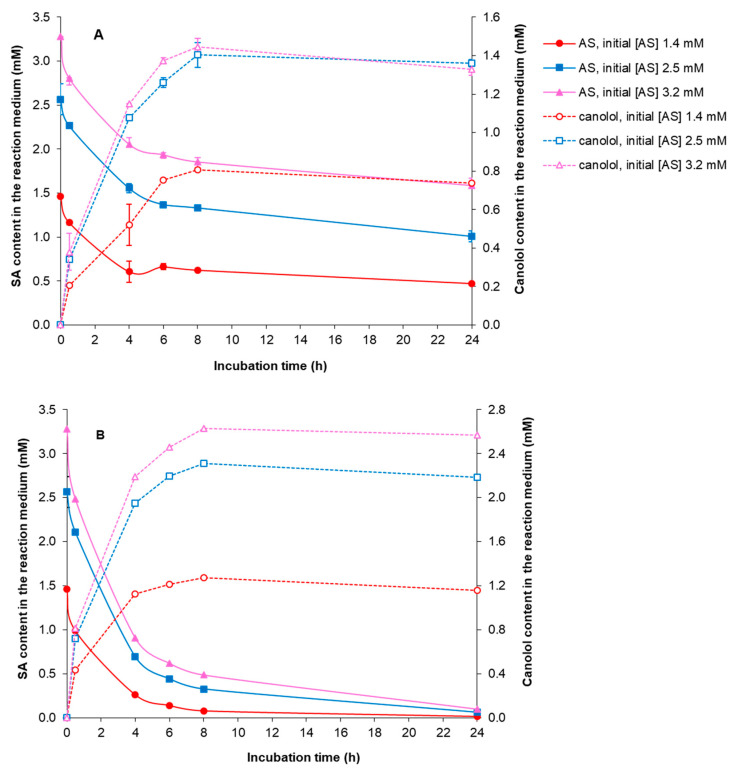
*Nle*PAD-mediated decarboxylation of commercial SA into canolol. The influence of the amount of *Nle*PAD on the decarboxylation of commercial SA into canolol was studied in relation to the initial SA concentration in the reaction medium. (**A**): 0.14 U PAD, (**B**): 0.30 U *Nle*PAD (activity determined in standard conditions). Incubations were carried out at 37 °C and pH 6, in a final volume of 0.35 mL. Assays were performed in duplicate.

First, the aqueous-phase bioconversion of SA into canolol was studied using 0.14 and 0.30 U *Nle*PAD in 0.35 mL of reaction medium (i.e., 0.4 and 0.86 U per mL of reaction medium, respectively), by varying the initial concentration of commercial SA (Figure 10). The reaction was carried out for 24 h. Whatever the quantity of *Nle*PAD and SA concentration tested, SA progressively disappeared as canolol was produced. The higher the initial SA concentration, the higher the amount of canolol produced. In our assay conditions, the maximal concentrations of canolol were obtained after 8 h in the presence of 0.30 U *Nle*PAD, with 1.276 (±0.002), 2.310 (±0.035) and 2.631 (±0.002) mM canolol produced from initial commercial SA concentrations of 1.4, 2.5 and 3.2 mM, respectively, which corresponded to a molar yield of bioconversion ranging from 82 to 92%. In the absence of *Nle*PAD, no SA bioconversion was observed.

Second, the bioconversion of biosourced SA into canolol in aqueous phase was evaluated using RSM as an SA-rich natural biomass substrate. It is worth noting that the SA in RSM is overwhelmingly present in esterified forms (mainly sinapoyl choline and sinapoyl glucose [2]) and that we previously showed that the fungus *N. lepideus* was unable to directly biotransform these esterified forms of SA into canolol in vivo [41]. In a previous study, we set up an in vivo two-step process to produce canolol from RSM, using, in a first step, the *An*FaeA enzyme, which was able to release free SA from the raw meal by hydrolyzing its conjugated forms [41]. Remarkably, *An*FaeA and the *Nle*PAD described in the current work display compatible temperature and pH ranges of activity: for *An*FaeA, 30–60 °C and pH 5–7 (with an optimum at 55 °C and pH 5–6) [42], and for *Nle*PAD, 30–55 °C and pH 5–7.5 (with an optimum at 37–45 °C and pH 6–6.5). Both enzymes (*An*FaeA from *A. niger* BRFM451 and *Nle*PAD from *N. lepideus* BRFM15) therefore emerged as promising candidates to release and decarboxylate SA from RSM as substrate and were thus implemented in in vitro processes.

**Figure 11:**
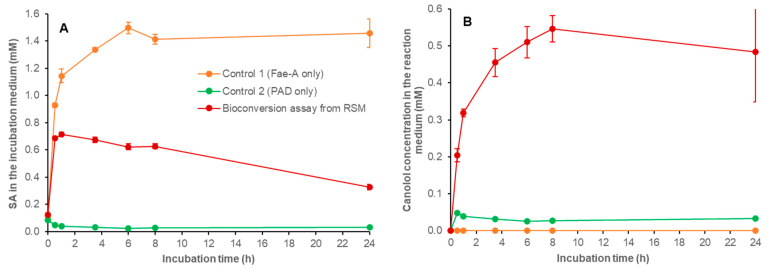
Bioconversion of biosourced SA from RSM into canolol in a one-step process. The formation and disappearance of aromatic compounds in the one-step process for *Nle*PAD-mediated bioconversion of biosourced SA from RSM was followed. Time-course of: (**A**) SA and (**B**) canolol. The reaction medium, consisting of 60 mg RSM, 39 nkat *An*FaeA per gram of RSM and 1.714 U purified *Nle*PAD, was incubated in 1 mL of 100 mM sodium phosphate buffer (pH 6) at 37 °C under agitation for 24 h. Assays were performed in duplicate.

The one-step process for bioconversion of SA from RSM into canolol was tested in the presence of both *An*FaeA and *Nle*PAD added together in the reaction mixture containing 6% (*w*/*v*) RSM. Since the beginning of the reaction, the release of free SA was observed with an optimum peak of 0.716 (±0.016) mM after 1 h of incubation, followed by a plateau of about 0.62–0.67 mM up to 8 h (Figure 11A). The release of free SA was concomitant with the synthesis of canolol, which reached a maximal concentration of 0.547 (±0.036) mM after 8 h (Figure 11B). The one-step process was thus effective and enabled the synthesis of about 10.3 µmol (1.86 mg) canolol per gram of initial RSM (DDM). In the control-1 reaction medium containing *An*FaeA only, we verified and quantified the RSM-hydrolyzing activity of *An*FaeA. In this case, the free SA released accumulated in the medium and reached up to 1.496 (±0.041) mM after 6 h of incubation (Figure 11A). In the control-2 reaction medium containing *Nle*PAD only, we observed traces of free SA (0.085 ± 0.003 mM) that corresponded to solubilization of the small fraction of SA present in the free form in RSM [49]. In this case, only traces of canolol were synthesized (0.048 ± 0.001 mM) (Figure 11B), thus confirming that *Nle*PAD was unable to directly biotransform esterified forms of SA.

It is worth noting that the release of SA from RSM could decrease the pH of the reaction medium down to 5–5.5, thus inhibiting *Nle*PAD activity (see Figure 7B: e.g., *Nle*PAD activity on SA was half as high at pH 5.5 as at pH 6.. In addition, one could also hypothesize that meal residues may have an inhibitory effect on *Nle*PAD activity. Therefore, we tested a two-step enzymatic bioconversion process from RSM to canolol.

**Figure 12:**
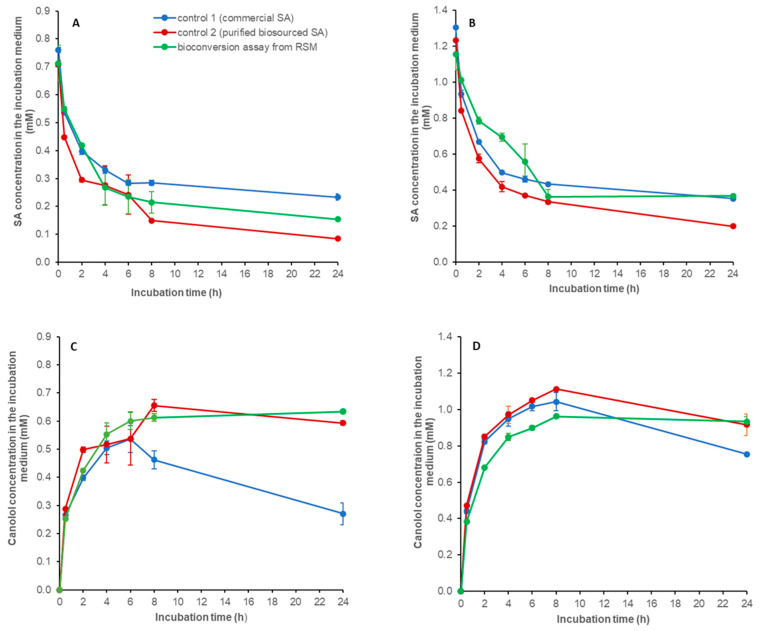
Bioconversion of biosourced SA from RSM into canolol in a two-step process. The formation and disappearance of aromatic compounds in the two-step process for *Nle*PAD-mediated bioconversion of biosourced SA from RSM was followed. Time-course of: (**A**) SA in bioconversion from 6% RSM (*w*/*v*), (**B**) SA in bioconversion from 12% RSM (*w*/*v*), (**C**) canolol in bioconversion from 6% RSM (*w*/*v*) and (**D**) canolol in bioconversion from 12% RSM (*w*/*v*). The reaction medium, consisting of 200 µL of the purified *Nle*PAD (0.343 U) and 200 µL of SA-containing solutions, was incubated at 37 °C under agitation for 24 h. Assays were performed in duplicate.

The two-step process of bioconversion of SA from RSM into canolol was tested by adding, successively and separately, *An*FaeA in the first step and *Nle*PAD in the following second step. Two initial concentrations of RSM in 100 mM sodium phosphate buffer (pH 6) were tested: 6 and 12% *w*/*v*. In the first step, *An*FaeA enabled the release of 1.354 (±0.019) and 2.415 (±0.014) mM free SA from 6% and 12% *w*/*v* initial RSM suspensions, respectively, which corresponded to 23.24 (±0.311) and 18.86 (±0.095) µmol of SA released per gram of initial RSM (expressed as grams of DDM). Both the resulting solutions of free biosourced SA were adjusted to pH 6 with NaOH (the release of SA decreases the pH of the reaction medium up to 5–5.5) then incubated in the presence of *Nle*PAD (200 µL of *Nle*PAD and 200 µL of the SA-containing solution). In both cases, we observed that SA concentration decreased as canolol concentration increased over the course of the incubation time (Figure 12). The higher the initial SA concentration, the higher the amount of canolol produced. In our assay conditions, the maximal concentrations of canolol were obtained after 8 h, with 0.613 (±0.014) and 0.961 (±0.003) mM canolol produced from initial SA concentrations of 0.711 (±0.066) and 1.155 (±0.086) mM, respectively (Figure 12C,D), corresponding to a molar yield of bioconversion of 86% and 83%, respectively. The two-step process was thus effective and enabled the synthesis of about 15–21 µmol canolol (2.7–3.8 mg) per gram of initial RSM (DDM). In the control-1 reaction medium, we verified that *Nle*PAD exhibited activity on similar initial concentrations of commercial SA, i.e., starting from 0.761 (±0.009) or 1.305 (±0.007) mM initial concentrations. In this case, the maximal canolol concentrations peaked at 6–8 h, with 0.536 (±0.048) and 1.043 (±0.051) mM canolol produced from initial concentrations of 0.761 and 1.305 mM commercial SA, respectively (which corresponded to a molar yield of bioconversion of 70 and 80%, respectively) (Figure 12). In the control-2 reaction medium, we tested *Nle*PAD activity on biosourced SA previously extracted and purified from enzymatically-hydrolyzed RSM, dried to powder and then redissolved in sodium phosphate buffer, starting from concentrations of 0.708 (±0.013) and 1.235 (±0.009) mM (Figure 12). In this case, the maximal concentrations of canolol produced were obtained after 8 h, with 0.655 (±0.021) and 1.112 (±0.001) mM canolol, respectively (which corresponded to a molar yield of bioconversion of 92.5 and 90%, respectively). 

## 4. Discussion

Phenolic-modifying microbial enzymes, particularly fungal enzymes, are often involved in both the biosynthesis and detoxification of compounds that have an aromatic structure. Consequently, they have the greatest potential for modifying plant-based substrates that have an aromatic structure. However, the conditions required to implement these enzymes are sometimes quite far from the conditions of industrial applications still today. Several ways can then be envisaged to improve the biotechnological capacities of these enzymes. The most immediate pathway is to explore natural biodiversity, and notably fungal biodiversity [54], in an attempt to isolate new enzymes with novel catalytic and technological properties (e.g., stability, production rate, kinetic and physico-chemical parameters, or particular substrate specificities as is the case here). The recent exponential surge in -*omics* data on filamentous fungi now opens up a wide field of in silico exploratory work. We were thus able to find sequences encoding putative PADs in annotated publicly-available basidiomycete genomes [39], including the species *N. lepideus*, *Schizophyllum commune* and *Stereum hirsutum*. It should be emphasized that these protein sequences did not show more than 45% similarity with the bacterial and yeast PAD sequences known to date, and no more than 75% between them. Among all of the species that we have previously screened in culture, only *N. lepideus* was found to be able to biotransform SA into canolol in vivo. The BRFM15 strain showed the highest substrate specificity towards SA compared to the other *p*-hydroxycinnamic acids described to date [41], thus highlighting a new metabolic feature of this fungal species. The brown-rot fungus *N. lepideus* (class Agaricomycetes, order Gloeophyllales) is an edible fungus with a particular aromatic compound metabolism that has historically attracted attention for its ability to grow on railroad ties and to tolerate creosote (a polycyclic aromatic hydrocarbon that was used to preserve woody materials) and its capacity for *O*-methylation/methoxylation of cinnamic acid [55,56]. More recently, *N. lepideus* has been described as having the ability to ferment xylose and lactose into ethanol, which is uncommon in filamentous fungi, especially in Basidiomycetes [57,58]. We have previously shown that *N. lepideus* was the only known species capable of biotransforming both FA into 4-VG and SA into canolol by non-oxidative decarboxylation [40]. Moreover, under our experimental conditions, this decarboxylation pathway was largely predominant compared to the β-oxidation-type pathway that led to the conversion of FA into vanillic acid and SA into syringic acid [40]. This metabolic capacity made it possible to obtain canolol in quantities of up to 1–1.5 g.L^−1^ in culture medium, which is favorable to industrial scale-up. The older literature on wood-rot fungi of the class Agaricomycetes described rather very favorable metabolic pathways for the bioconversion of *p*-hydroxycinnamic acids into the corresponding *p*-hydroxybenzoic acids, aldehydes or alcohols [59], via a β-oxidation-type pathway. For example, the species *Pycnoporus cinnabarinus* very efficiently biotransformed FA into vanillin and vanillic alcohol and pAC into *p*-hydroxybenzaldehyde [59,60]. To our knowledge, the non-oxidative decarboxylation pathway of the same *p*-hydroxycinnamic acids has never been described in this type of fungus. 

In the current work, we found a sole protein sequence in the intracellular proteome of the *N. lepideus* strain BRFM15 that corresponded to a PAD sequence. The N-terminus and four internal peptide sequences of this protein matched the sequence of the protein predicted as a PAD from the publicly-available genome of *N. lepideus* strain HHB14362 (Prot Id KZT30061 in the NCBI database). Both the C- and the N-terminal sequences of bacterial PADs appeared to play a crucial role in the activity and substrate specificity of the enzyme [25,61]. Interestingly, both the N-terminal and C-terminal amino acid sequences of *Nle*PAD did not share more than 65% similarity with the sequences of the PADs from *S. commune*, *S. hirsutum*, *I. farinosa* and *A. luchuensis*. The *Nle*PAD, purified from a crude intracellular extract of *N. lepideus* BRFM15 grown in the presence of SA as PAD-inducer, was shown to be homodimeric, with an apparent molecular mass of 2 × 22 kDa. The enzyme was active on both SA and FA with activities of the same range under our experimental conditions. For bacterial and yeast PADs, the optimum temperature is between 20 °C and 45 °C, and the optimum pH is between 4 and 7.3 [9,20,22,32,62,63,64,65]. Using FA and SA as substrates, the *Nle*PAD displayed comparable biochemical characteristics to those described for bacterial and yeast PADs, with a temperature range of activity between 30 °C and 55 °C and an optimum between 37 °C and 45 °C, and a pH range of activity between 5 and 7.5 with an optimum of 6–6.5. However, these characteristics were slightly different from those of other filamentous fungal PADs. For instance, the PAD from the ascomycete *Isaria farinosa* showed optimal activity at temperatures of 14–19 °C [36], and the PAD from the basidiomycete *S. commune* remained highly stable at alkaline pH 11 [38]. The *Nle*PAD was highly stable at 4 °C and at pH values between 6 and 8 even after several days. It retained ~80% and 50% of activity after incubation at 55 °C for 1 h and 10–12 h, respectively, thus conferring good thermostability compared to yeast and bacterial PADs for which residual activity was less than 50% (from 0 to 47% according to the microorganism) after incubation at 50–55 °C for 30–60 min [24,62,64,66]. The *Nle*PAD thus displayed a relatively broad temperature and pH stability for an intracellular enzyme, enabling flexible incubation conditions with only little loss of activity. 

In the case of PAD activity, the kinetic parameters remained tricky to compare, in absolute values, with data from literature, as the activity assays used varied widely in terms of method used (spectrophotometric or HPLC assays) and the composition of the mixture assay (type and concentration of substrate, pH, temperature, incubation time). In the case of bacterial and yeast PADs, specific activities, K_M_ and V_max_ have mainly been measured for pCA, FA and CafA. Taking 100% as baseline activity measured on pCA, the following relative ratios of specific activities on pCA, FA and CafA, respectively, were found: 100:75:34 for *Bacillus licheniformis* PAD [24], 100:70.4:31.6 for *B. atrophaeus* PAD [65], 100:77:100 for *Lactobacillus brevis* PAD [22], and 100:88.5:7.6 for *Candida guilliermondii* PAD [64]. For *Nle*PAD, V_max_ and k_cat_ were about 6-fold higher for FA than for SA, showing that the catalytic cycle duration was shorter for FA than for SA. However, the enzyme showed the same affinity for both substrates. In the case of *B. licheniformis* PAD, an activity on SA could be detected, but the specific activity on SA was 343- and 256-fold lower than the specific activity on pCA and FA, respectively [24]. Moreover, none of the PADs described in filamentous fungi to date showed activity on SA [36,37,38]. In light of these data, *Nle*PAD emerged as an outstanding candidate for the in vitro bioconversion of SA into canolol. 

Several authors have previously reported inhibition/deactivation of bacterial PADs by the substrate and product of the enzymatic reaction [19,67,68]. pHCAs, such as FA and SA, and the corresponding vinyl derivatives such as 4-VG and canolol, are poorly soluble in water. However, to carry out inhibition tests on PAD activity, it is mandatory to test suitable ‘high’ concentrations of substrates and products of the enzymatic reaction, which involves solubilization in water-miscible solvents, such as ethanol, methanol or acetonitrile. In the current work, ethanol, methanol and acetonitrile had a strong inhibitory effect on *Nle*PAD activity, even at low concentrations, which prevented this type of study from being performed.

In order to unveil what makes *Nle*PAD special, we carried out a comparative structural analysis of *Nle*PAD and *Bsu*PAD, which revealed some striking differences in the neighborhood of both the carboxylic acid moiety-binding tyrosines and the catalytic Arg/Glu dyad. Among these, the drastic Val → His mutation in the vicinity of the Arg/Glu dyad (H100 in *Nle*PAD) seems to hold a functional importance since it is conserved in all analyzed fungal PADs (Figure 3, His indicated in yellow boxed letter) but cannot explain on its own the activity on SA, as these other fungal PADs are not active on SA. One other important mutation observed only in *Nle*PAD is the Leu → Met mutation on the tyrosine side (L72 in *Bsu*PAD and M81 in *Nle*PAD, Met indicated in yellow boxed letter on Figure 3), which may alter the H-bond network/interaction between the substrate and the enzyme active site. All in all, the acquisition of this new substrate specificity by *Nle*PAD is probably the result of multiple natural mutations that warrant being tested by directed mutagenesis.

Turning our attention towards the implementation of *Nle*PAD into an applied process, we envisioned using RSM as a biological and renewable feedstock of SA to serve as the precursor to canolol by enzymatic bioconversion. A chemoenzymatic process, based on a *B. pumilus* PAD, engineered to gain SA-decarboxylating activity, was developed by Morley et al. [33] to produce canolol from free SA obtained after alkaline hydrolysis of RSM and solvent extractions for purification and concentration. The bioconversion of SA into canolol was carried out in a biphasic aqueous buffer/toluene system. This two-step process made it possible to synthesize 3 mg canolol per gram of initial raw meal [33]. Taking these considerations forward, here we aimed to bring proof-of-concept for the in vitro enzymatic synthesis of canolol from biosourced free SA released from RSM in aqueous media. In our current study, the native *Nle*PAD first demonstrated in vitro effectiveness as a biocatalyst for the synthesis of canolol from commercial SA in an aqueous medium, with a molar yield of 92% in the best conditions studied. Then, we showed that the combination of the two enzymes in vitro was effective for releasing SA from RSM and decarboxylating it into canolol in aqueous media, both by the one-step and two-step processes tested here. To our knowledge, this is the first time that *An*FaeA and a fungal PAD have been applied to RSM as raw natural substrate. In our processes, the enzymatic decarboxylation of SA extracts from RSM in aqueous media led to an overall yield of about 1.9–3.8 mg canolol per gram of RSM (DDM), i.e., about 4.2 times more than the yield obtained by physico-chemical treatments [28,29,30,31]. 

## 5. Conclusions

The native enzyme *Nle*PAD showed an unprecedented SA-decarboxylating activity and seems to be very promising as a new biotechnological tool to generate biobased vinylphenols of industrial interest, especially canolol, a valuable platform chemical for health, nutrition, cosmetics and green chemistry. The process described here, based on the sole use of enzymes in an aqueous medium and mild conditions, opens new perspectives for the synthesis of valuable vinylphenols from renewable biomasses such as oilseed meals. This work thus lays the foundation for a new set of challenges to scale up the process by improving its overall yield and productivity without using organic solvents. Potential routes forward could be immobilizing and recycling the enzymes and/or adding an appropriate adsorbent to the aqueous reaction medium to continuously harvest the canolol produced. Furthermore, the separate production of two different enzymes, by two types of fungi such as *A. niger* and *N. lepideus*, which show very different physiology and cultivation times in bioreactors, could be an obstacle for scaling up. Therefore, the heterologous production of an *An*FaeA–*Nle*PAD chimeric protein in the *Aspergillus niger* host could be considered. This type of chimera (for example, *An*FaeA–xylanase or laccase–cellulose binding module), based on the principle of bacterial cellulosomes, has already shown its effectiveness for the deconstruction of plant biomasses [69], and thus holds great promise for further improving the bioproduction of canolol from bioresources. 

## Figures and Tables

**Table 1:** Purification of *Nle*PAD.

Purification Step	Volume (mL)	Protein Concentration (mg.mL^−1^)	Activity ^b^ (U.mL^−1^)	Total Activity ^b^ (U)	Specific Activity ^b^ (U.mg Proteins^−1^)	Yield (%)	Purification (-Fold)
Crude extract	140	2.075	57.1	7995	27.52		
DEAE Sepharose Fast Flow ^a^	14	5.472	440.9	6172	80.57	77	2.9
Sephacryl S-100HR ^a^	1.55	3.279	1819.0	2819	554.70	35	20.1
Superdex 75 Prep Grade ^a^	4	0.204	441.8	1767	2161.78	22	78.6

^a^ After concentration with a 10-kDa polyethersulfone membrane (Sartorius Stedim Biotech, Goettingen, Germany). ^b^ Determined with FA as substrate.

**Table 2:** Biochemical and kinetic characteristics of *Nle*PAD.

	Substrate	
	Sinapic Acid	Ferulic Acid
Temperature range of activity	30–50 °C	30–55 °C
Optimal temperature	37 °C	45 °C
Temperature stability		
Half-life (h) at 4 °C	>120	>120
30 °C	91.6	90
37 °C	64.6	58
45 °C	28.2	18.3
55 °C	12.4	10.2
pH range of activity	5.5–7.5	5.0–7.5
Optimal pH	6	6.0–6.5
pH stability		
Residual activity after 2 days (%)		
pH 4	16	12
pH 5	72	81
pH 6	88	77
pH 7	97	97
pH 7.5	90	91
pH 8	95	100
Residual activity after 7 days (%)		
pH 4	1	1
pH 5	31	37
pH 6	70	63
pH 7	86	90
pH 7.5	84	84
pH 8	80	92
K_M_ (mM)	3.9	2.6
V_max_ (U.mg^−1^)	600	3735
k_cat_ (s^−1^)	6.3	39.2
k_cat_/K_M_ (s^−1^.mM^−1^)	1.6	14.8

## Data Availability

The datasets supporting the conclusions of this article are included within the article or the additional files (Additional File 1: Figures S1–S8, Additional File 2: Table S1).

## Supplementary Materials

The following supporting information can be downloaded at: https://www.mdpi.com/article/10.3390/bioengineering11020181/s1. Additional File 1: Figure S1. Sequence of the gene encoding *N. lepideus* HHB14362 phenolic acid decarboxylase and the deduced protein (Protein Id 1126845) predicted from the genome annotation [39]. The sequence has been deposited in the NCBI database with the number KZT30061.1. Figure S2. Purification of *Nle*PAD by size exclusion chromatographies (SEC). Figure S3. Identification of 4-vinylcatechol produced via the decarboxylation of caffeic acid by *Nle*PAD. HPLC elution profiles of phenolic compounds detected in a reference reaction medium in the absence of *Nle*PAD (A) and in the presence of *Nle*PAD (B), and corresponding mass spectra of caffeic acid and 4-vinylcatechol. Figure S4. UV-VIS spectra of canolol (A), 4-vinylguaiacol (4-VG) (B), and 4-vinylphenol (4-VP) (C) detected in the *Nle*PAD-catalyzed bioconversion mixtures from sinapic acid (SA), ferulic acid (FA) and *p*-coumaric acid (pCA), respectively. Comparison with spectra from canolol, 4-VG and 4-VP standards. Figure S5. Confidence scores of AlphaFold2 structural prediction of *Nle*PAD. Figure S6. Surface patches of PADs. Figure S7. View of the proposed active site “entrance” of PADs. Figure S8. Neighborhood analysis of substrate-binding tyrosines and catalytic residues of PADs. Additional File 2: Table S1. List of the proteins detected and identified in the proteome of *N. lepideus* BRFM15 grown in the presence of sinapic acid as PAD inducer, in comparison with a reference culture (without any inducer).

## Abbreviations

CafA	caffeic acid
pCA	*p*-coumaric acid
DDM	defatted dry matter
FA	ferulic acid
pHCA	*p*-hydroxycinnamic acid
LC-MS/MS	liquid chromatography–tandem mass spectrometry
PAD	phenolic acid decarboxylase
PES	polyethersulfone
PMSF	phenylmethanesulfonyl fluoride
RSM	rapeseed meal
SA	sinapic acid
SEC	size exclusion chromatography
TCA	trichloroacetic acid
4-VG	4-vinylguaiacol
4-VP	4-vinylphenol
