# Supplementary material for: A New Phenolic Acid Decarboxylase from the Brown-Rot Fungus Neolentinus lepideus Natively Decarboxylates Biosourced Sinapic Acid into Canolol, a Bioactive Phenolic Compound
Source: Bioengineering (Basel). 2024 Feb 14;11(2):181. doi: 10.3390/bioengineering11020181 (PMC10886158; doi:10.3390/bioengineering11020181)
Supplement: Supplementary file 1 [file bioengineering-11-00181-s001.zip › Additional File 1 VF Bioengineering-2851528.pdf]

Additional File 1

A new Phenolic Acid Decarboxylase from the Brown-Rot Fungus *Neolentinus lepideus* Natively Decarboxylates Biosourced Sinapic Acid into Canolol, a Bioactive Phenolic Compound

Elise Odinot [1], Alexandra Bisotto-Mignot [2], Toinou Frezouls [2], Bastien Bissaro [2], David Navarro [2], Eric Record [2], Frédéric Cadoret [2], Annick Doan [2], Didier Chevret [3], Frédéric Fine [4] and Anne Lomascolo [2,*]

[1] OléoInnov, 19 rue du Musée, F-13001 Marseille, France; elise.odinot@oleoinnov.com
[2] Aix-Marseille Université, INRAE, UMR1163 BBF Fungal Biodiversity and Biotechnology, 163 avenue de Luminy, F-13009 Marseille, France; alexandra.bisotto@gmail.com (A.B.-M.); bastien.bissaro@inrae.fr (B.B.); david.navarro@inrae.fr (D.N.); eric.record@inrae.fr (E.R.); cadfred86@gmail.com (F.C.); annick.doan@inrae.fr (A.D.)
[3] INRAE, UMR1319 MICALIS Institute, PAPPSO, Domaine de Vilvert, F-78350 Jouy-en-Josas, Cedex, France; didier.chevret@inrae.fr
[4] TERRES INOVIA, Parc Industriel, 11 rue Monge, F-33600 Pessac, France; f.fine@terresinovia.fr
* Correspondence: anne.lomascolo@univ-amu.fr; Tel.: +33-68-857-1660

Figure S1. Sequence of the gene encoding the *N. lepideus* HHB14362 phenolic acid decarboxylase and the deduced protein (Protein Id 1126845) predicted from the genome annotation [39]. The sequence has been deposited in the NCBI database with the number KZT30061.1.

Figure S2. Purification of *Nle*PAD by size exclusion chromatographies (SEC). Elution profile of proteins after gel filtration on a Sephacryl S-100HR column (**A**) followed by a Superdex 75 Prep Grade column (**B**). The fractions selected after each purification step are framed with dotted grey lines. SDS-PAGE analysis (**C**) after different purification steps (5 µg total proteins deposited for each lane). Lane 1: crude extract; lane 2: concentrated DEAE-Sepharose eluate; lane 3: Sephacryl S-100 HR eluate; lane 4: Superdex S75 eluate; M: molecular mass protein standards.

Figure S3. Identification of 4-vinylcatechol produced via the decarboxylation of caffeic acid by *Nle*PAD. HPLC elution profiles of phenolic compounds detected in a reference reaction medium in the absence of *Nle*PAD (A) and in the presence of *Nle*PAD (B), and corresponding mass spectra of caffeic acid and 4-vinylcatechol.

Figure S4. UV-VIS spectra of canolol (A), 4-vinylguaiacol (4-VG) (B), and 4-vinylphenol (4-VP) (C) detected in the *Nle*PAD-catalyzed bioconversion mixtures from sinapic acid (SA), ferulic acid (FA) and *p*-coumaric acid (pCA) respectively. Comparison with spectra from canolol, 4-VG and 4-VP standards.

Figure S5. Confidence scores of AlphaFold2 structural prediction of *Nle*PAD.

Figure S6. Surface patches of PADs.

Figure S7. View of the proposed active site “entrance” of PADs.

Figure S8. Neighborhood analysis of substrate-binding tyrosines and catalytic residues of PADs.

References cited in figure legends:

Frank A, Eborall W, hyde R, Hart S, Turkenburg JP, Grogan G. Mutational analysis of phenolic acid decarboxylase from Bacillus subtilis (BsPAD), which converts bio-derived phenolic acids to styrene derivatives. Catalysis Sci Technol. 2012; 2: 1568-1574. https://doi.org/10.1039/C2CY20015E.

Jumper J, Evans R, Pritzel A et al. Highly accurate protein structure prediction with AlphaFold. Nature. 2021; 596:583–589. https://doi.org/10.1038/s41586-021-03819-2.

Matte A, Grosse F, Bergeron H, Abokitse K, Lau PCK. Structural analysis of Bacillus pumilus acid decarboxylase, a lipocalin-fold enzyme. Acta Cryst Section F: Struct Biol Cryst Comm. 2010; F66: 1407-1414. https://doi.org/10.1107/S174430911003246X.

Odinot E, Fine F, Sigoillot JC, Navarro D, Laguna O, Bisotto A, Peyronnet C, Ginies C, Lecomte J, Faulds CB, Lomascolo A. A two-step bioconversion process for canolol production from rapeseed meal combining an *Aspergillus niger* feruloyl esterase and the fungus *Neolentinus lepideus*. Microorganisms. 2017;5:67, https://doi.org/103390/microorganisms5040067.

```
1    ATGAGCCACGAAGGAGCAACGTCAGAAGAGTTCAAGCAGATTGAAGGGAAGCGGTTCAAGTACACGTATGGTTTG
      M  S  H  E  G  A  T  S  E  E  F  K  Q  I  E  G  K  R  F  K  Y  T  Y  G  L
1    GGATGGACGTATGAGGTCAGCAGAGCCATCTCCCAGTACGCAAACCTTGACACACATGTTTGCAACAGATGTACT
      G  W  T  Y  E                Intron 1                               M  Y
76   TCCGCTCCTTAACGCGATGTGTATACCGGATTCTTTCGGGGCCTTTGGCGGGTAGAGTTAACTTCCAGCATGCGC
26   F  R  S  L  T  R  C  V  Y  R  I  L  S  G  P  L  A  G  R  V  N  F  Q  H  A
     ACTACCAGAAAATCAGAGACAACGTCTGGCAATGCAGCTGGCTCGAAGGTCAAGCATCTCATTCGCAGAATAACA
151  H  Y  Q  K  I  R  D  N  V  W  Q  C  S  W  L  E                Intron 2
     AAAGGCTGACACACTTCTCTCATGCAGAAACCGGACAGTAGTCTCCATGGTTGTCGACTTTGATCAACAGACTG
33                              E  T  G  T  V  V  S  M  V  V  D  F  D  Q  Q  T
     TCAAGACTTTCGCAACGTTCTCGAGAGGCCACTGGGATCTCCCCGATCAGGTGAGGTCTAAGCTACCCACCAGCA
226  V  K  T  F  A  T  F  S  R  G  H  W  D  L  P  D  Q
     AGGTATATCCGCTGACCCGTCGATGTTATCAGGCGAAAGGGTGGAAGCGCAATCCGGAGGACATGGCAAGATGGA
58       Intron 3                       A  K  G  W  K  R  N  P  E  D  M  A  R  W
301  GGACTCTCGCGCAGAAAGGAAAGGACCAGGCAGACAAGCATGTGCTAGTCGAACATGCGAAAATGTCCGAGTTGA
     R  T  L  A  Q  K  G  K  D  Q  A  D  K  H  V  L  V  E  H  A  K  M  S  E  L
74   CGAGCGGGCAGGGAGATCTGCCAGACATAGACGATTCTTGGGAGACCATGTAG
     T  S  G  Q  G  D  L  P  D  I  D  D  S  W  E  T  M  *
```

Figure S1. Sequence of the gene encoding the *N. lepideus* HHB14362 phenolic acid decarboxylase and the deduced protein (Protein Id 1126845) predicted from the genome annotation (https://genome.jgi.doe.gov/programs/fungi/index). The sequence has been deposited in the NCBI database with the number KZT30061.1.

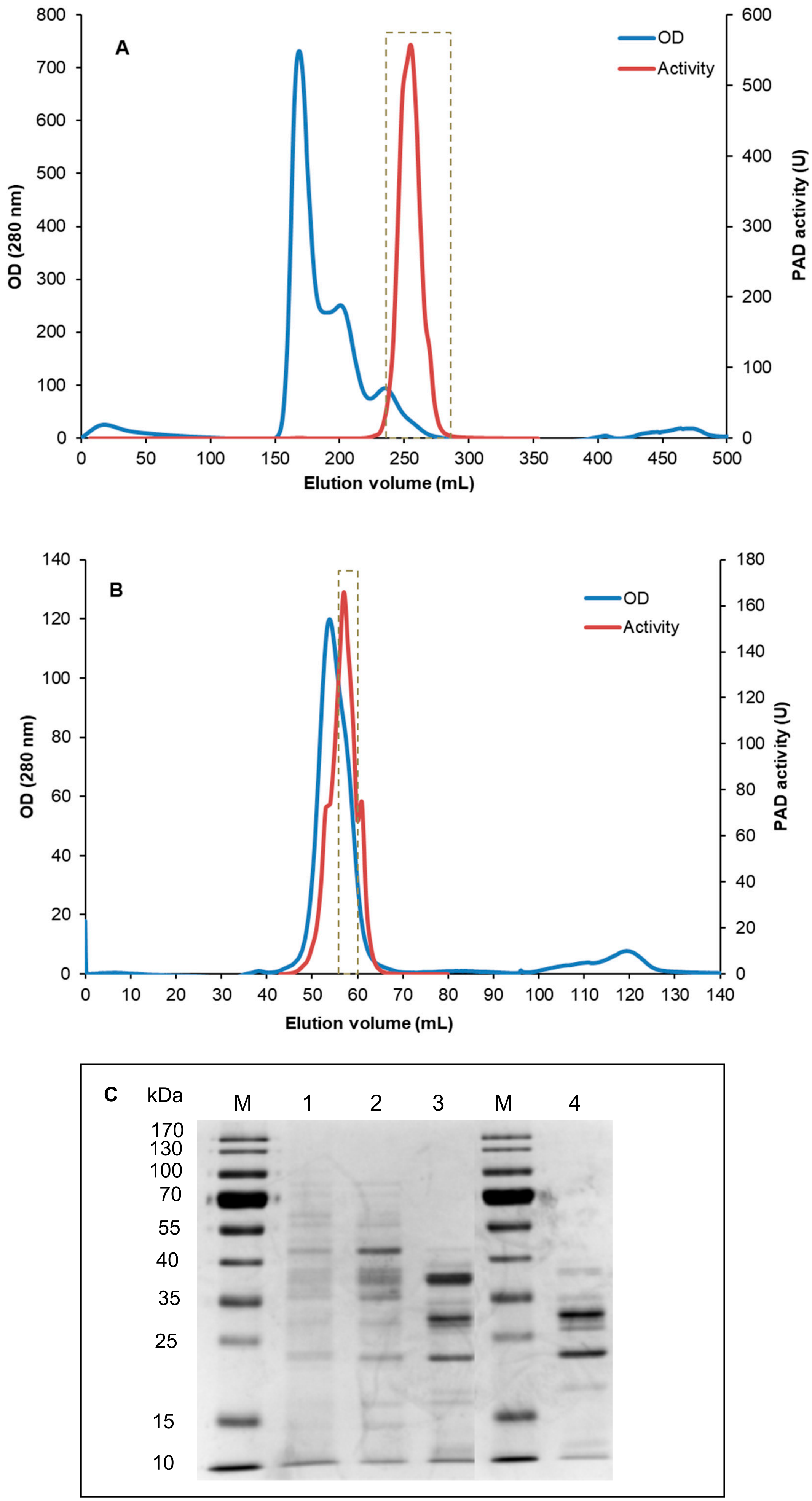
A
OD
Activity
OD (280 nm)
PAD activity (U)
Elution volume (mL)
B
OD
Activity
OD (280 nm)
PAD activity (U)
Elution volume (mL)
C
kDa
M 1 2 3 M 4
170
130
100
70
55
40
35
25
15
10

Figure S2. Purification of *Nle*PAD by size exclusion chromatographies (SEC). Elution profile of proteins after gel filtration on a Sephacryl S-100HR column (**A**) followed by a Superdex 75 Prep Grade column (**B**). The fractions selected after each purification step are framed with dotted grey lines. SDS-PAGE analysis (**C**) after different purification steps (5 µg total proteins deposited for each lane). Lane 1: crude extract; lane 2: concentrated DEAE-Sepharose eluate; lane 3: Sephacryl S-100 HR eluate; lane 4: Superdex S75 eluate; M: molecular mass protein standards. *Nle*PAD activity was measured in standard reaction conditions.

After the first SEC step (A), the area of the peak corresponding to the fractions containing *Nle*PAD activity represented 5.2% of the total area of the eluted proteins (OD_{280}). After the second SEC step (B), the area of the peak containing active fractions represented 36.7% of the total area of the eluted proteins (OD_{280}). The first step of SEC reduced therefore the contaminant protein content by about 95% while the following SEC eliminated about 67% of the remaining proteins, giving a roughly estimated degree of purity of 98% after all the purification steps.

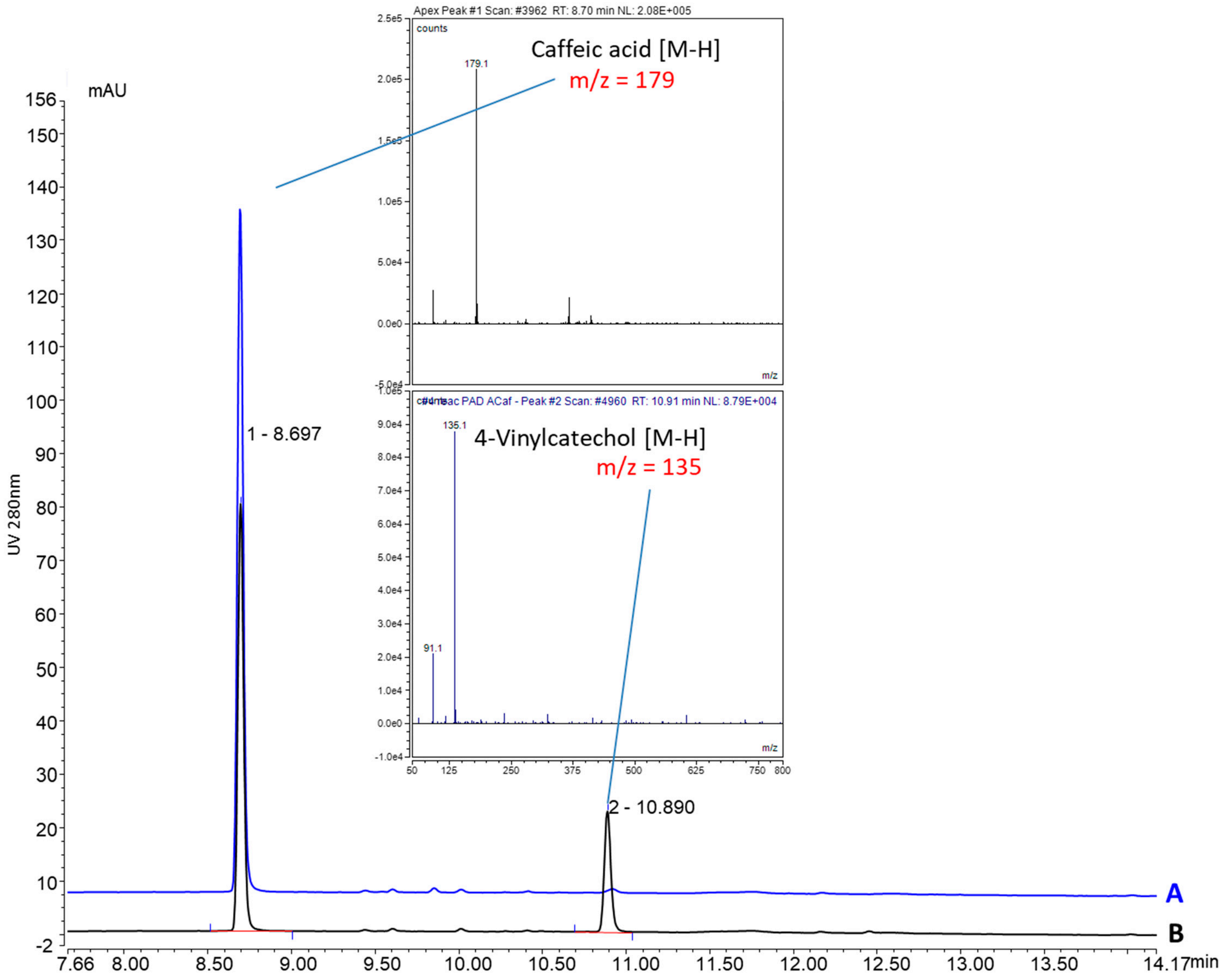

Figure S3. Identification of 4-vinylcatechol produced via the decarboxylation of caffeic acid by *Nle*PAD. HPLC elution profiles of phenolic compounds detected in a reference reaction medium in the absence of *Nle*PAD (**A**) and in the presence of *Nle*PAD (**B**), and corresponding mass spectra of caffeic acid and 4-vinylcatechol.

In this case, UHPLC analysis of monophenolics was performed at 280 and 310 nm on an Ultimate 3000RS model apparatus (Thermo Scientific) equipped with an automatic injector and a Diode Array Detector (DAD, Ultimate 3000, Thermo Scientific), coupled with an ISQ-EM Single Quadrupole Mass Spectrometer with Heated ESI-interface (Thermo Scientific). Separation was achieved on an Acquity BEH C18 reversed-phase column (1.7 µm, 2.1 × 150 mm, Waters, Guyancourt, France). The column temperature was maintained at 30 °C. The flow rate was 0.3 $mL.min^{-1}$. The used mobile phases were: solvent A (water acidified by 0.1% formic acid) and solvent B (acetonitrile 100%). The gradient changed as follows: solvent B started at

5% for 2 min, increased to 100% in 13 min; B was held at 100% for 7 min and then decreased to 5% until the end of running (32 min). In these experimental conditions, caffeic acid and 4-vinylcatechol were eluted at 8.7 min and 10.9 min, respectively. UHPLC-ESI-MS data acquisition was performed with the Chromeleon software v7.2.10 (Thermo Scientific). MS acquisition parameters were as follows: the heated ESI was operated at 172 °C in negative (at −2 kV) and positive (at +3kV) mode spray current, with a sheath gas pressure of 35.8 psig, an auxiliary gas pressure of 4.0 psig and a sweep gas pressure of 0.5 psig. The capillary temperature was 300°C. Masses from 50 to 800 m/z were monitored.

It is noteworthy that the identity of canolol and 4-VG, produced in cultures of *N. lepideus* BRFM15 supplemented with sinapic acid and ferulic acid respectively, was previously confirmed by GC-MS analysis as described by Odinot et al. (2017).

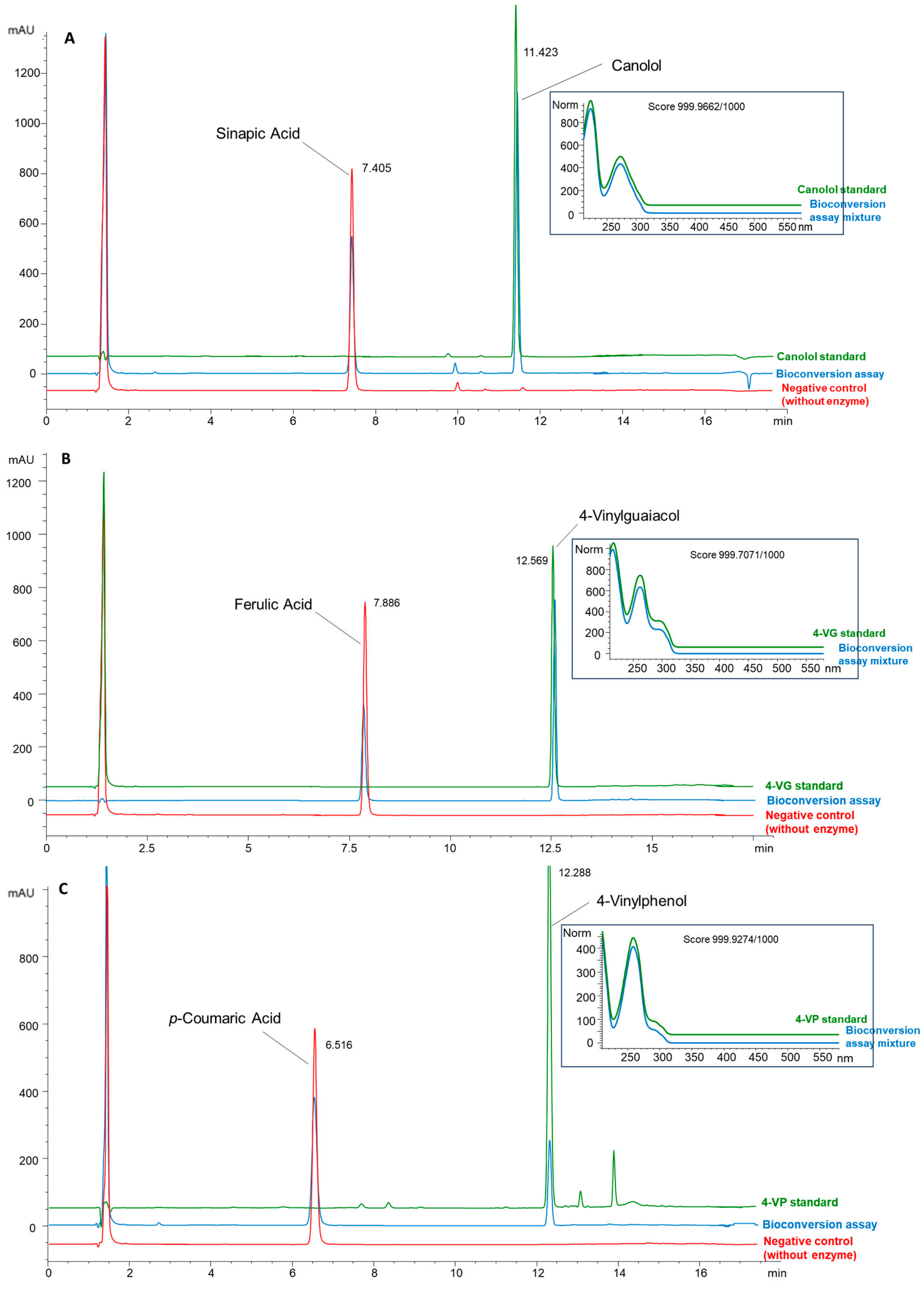

Figure S4. UV-VIS spectra of canolol **(A),** 4-vinylguaiacol (4-VG) (**B**), and 4-vinylphenol (4-VP) **(C)** detected in the *Nle*PAD-catalyzed bioconversion mixtures from sinapic acid (SA), ferulic acid (FA) and *p*-coumaric acid (pCA) respectively. Comparison with spectra from canolol, 4-VG and 4-VP standards.

In this case, HPLC analysis of monophenolics was performed at 30°C on a model Agilent1260 apparatus (Agilent Technologies, Massy, France) equipped with a diode array detection system and a 100-position autosampler autoinjector. Separation was achieved on a C18 reversed-phase column (Agilent Eclipse Plus column, 3.5 µm, 4.6 × 100 mm). The flow rate was 1 $mL.min^{-1}$. The mobile phases used were solvent A: water acidified by 0.05% phosphoric acid and acetonitrile (95:5, v/v), and solvent B: acetonitrile 100%. The gradient program was as follows: 10% B for 4 min, 10% B to 40% B (6 min), 40% B to 90% B (3 min), 90% B for 2 min, 90% B to 10% B (1 min), 10% B for 2 min. Total run time was 18 min. The injection volume for all samples was 5 µL. Phenolic compounds were monitored separately at 220 nm. Additionally, UV/Vis spectra were recorded in the range of 200–600 nm. The Agilent1260 ChemStation processed the data, and the identification was performed by external standard calibrations.

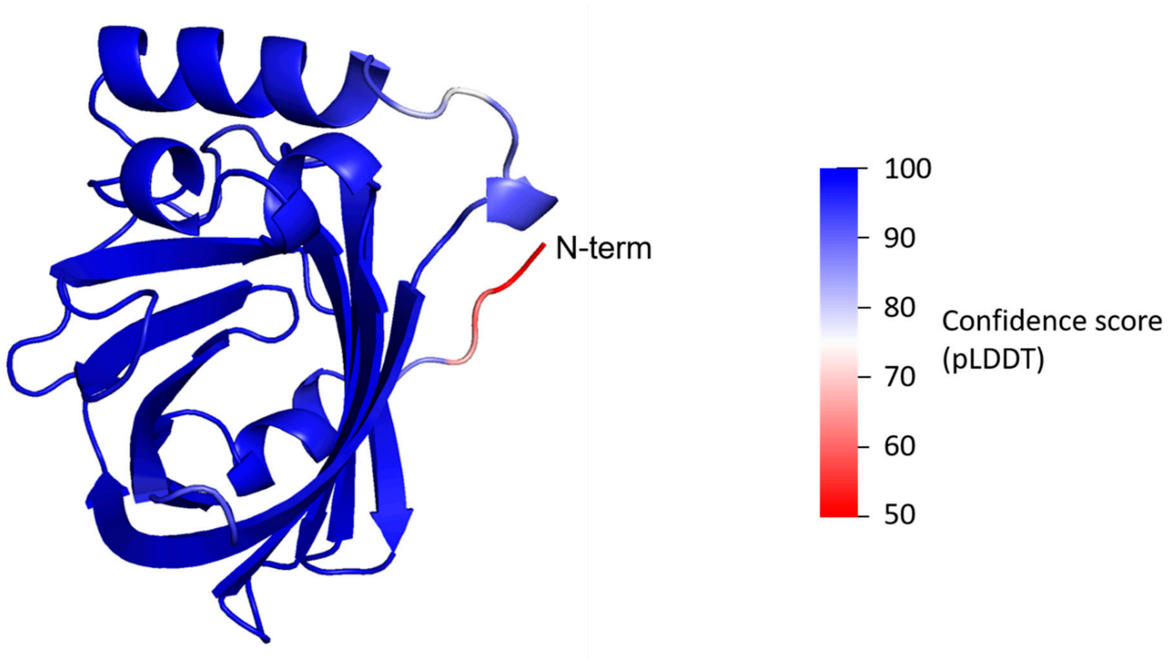

Figure S5. Confidence scores of AlphaFold2 structural prediction of *Nle*PAD.

The figure shows the per-residue confidence score (pLDDT) values mapped on the AlphaFold2 structural prediction (Jumper et al. 2021) of *Nle*PAD shown as cartoon. This structure has been used to compute the surface patches shown in **Figures S6 and S7**. The color scale is provided on the right-hand side of the figure. Dark blue colors indicate very high confidence scores (i.e. pLDDT values > 90). pLDDT scores < 70 are considered as low. pLDDT is a measure of local accuracy and corresponds to the model's prediction of its score on the local Distance Difference Test.

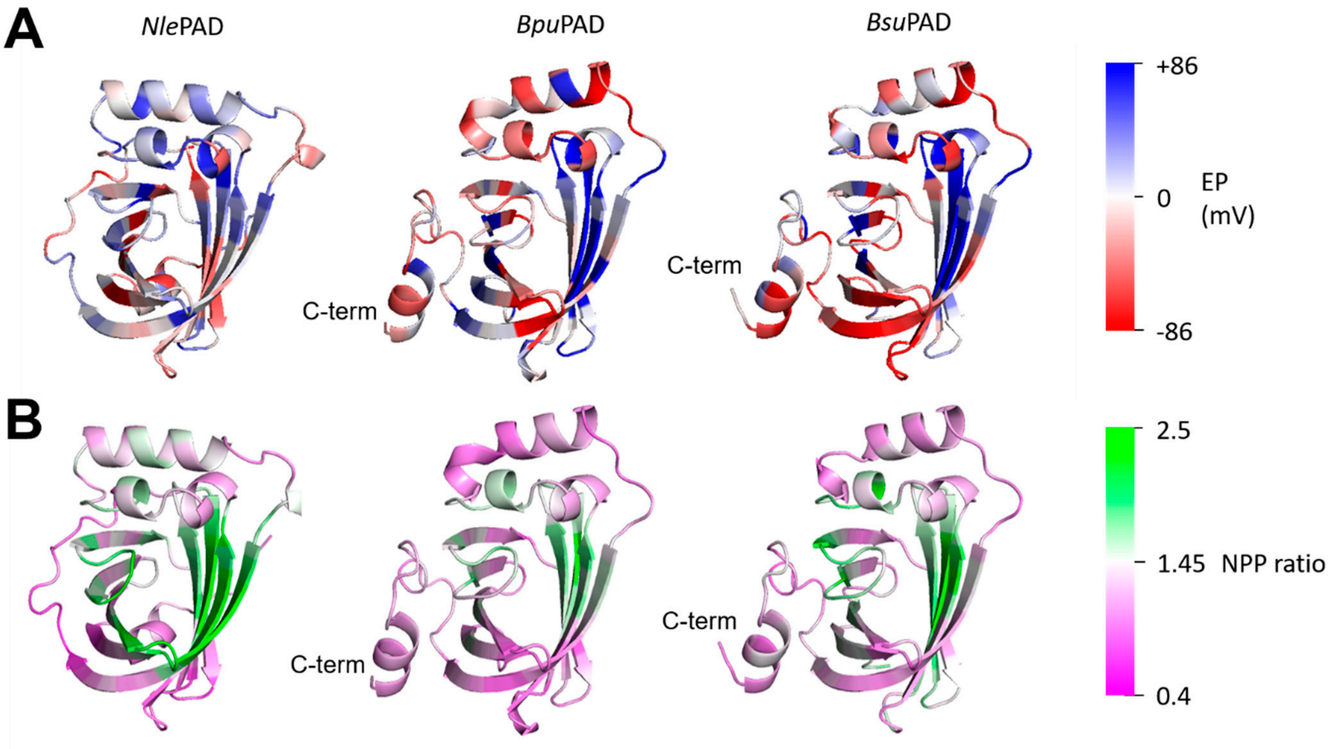

Figure S6. Surface patches of PADs.

The figure shows **(A)** the electrostatic potential (EP) and **(B)** the non-polar/polar (NPP) ratio for *Nle*PAD (AlphaFold2 model), *Bpu*PAD (PDB code 3NAD; Matte et al. 2010), and *Bsu*PAD (PDB code 4ALB; Frank et al. 2012) shown as cartoon. The color scales provided on the right-hand side indicate the EP from negative charge (red) to positive charge (blue) in panel A, and the NPP ratio, from 0.4 (more polar; magenta) to 2.5 (more non-polar; green) in panel B.

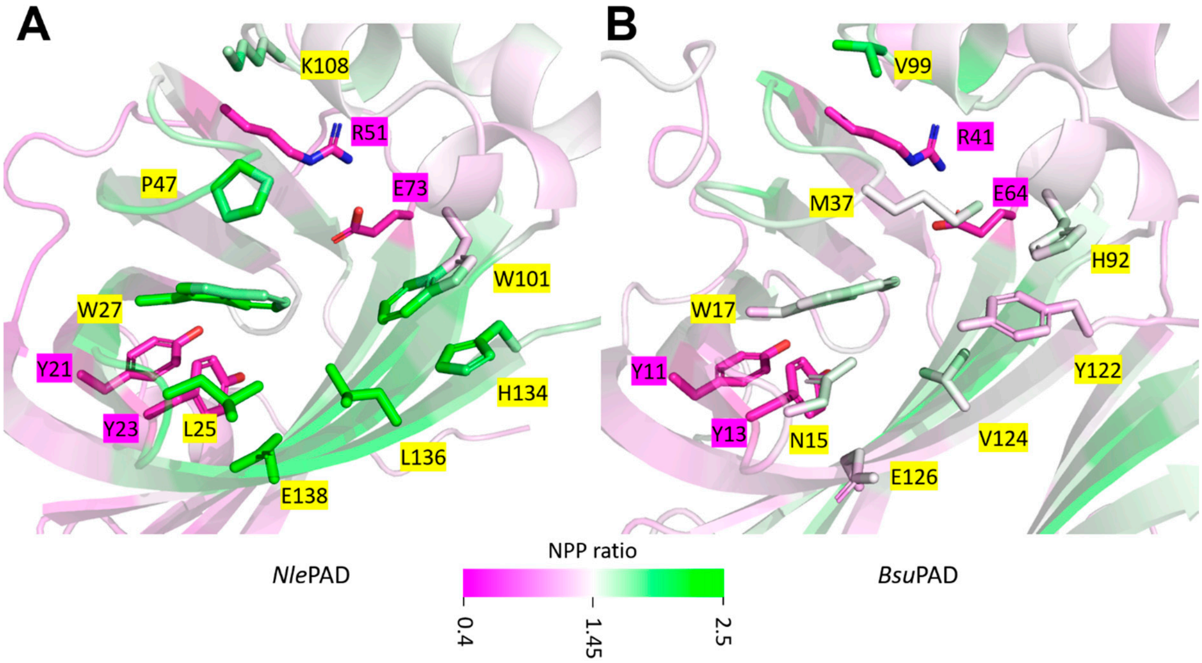

Figure S7. View of the proposed active site "entrance" of PADs.

The figure shows the structures of **(A)** *Nle*PAD (AlphaFold2 model; Jumper et al. 2021) and **(B)** *Bsu*PAD (PDB code 4ALB; Frank et al. 2012), shown as cartoon and colored according to the non-polar/polar (NPP) ratio (see **Figure S11B**). Catalytic residues (R51/R41 and E73/E64 in *Nle*PAD/*Bsu*PAD) and substrate-binding tyrosines (Y21/Y11 and Y23/Y13) are shown as magenta sticks.

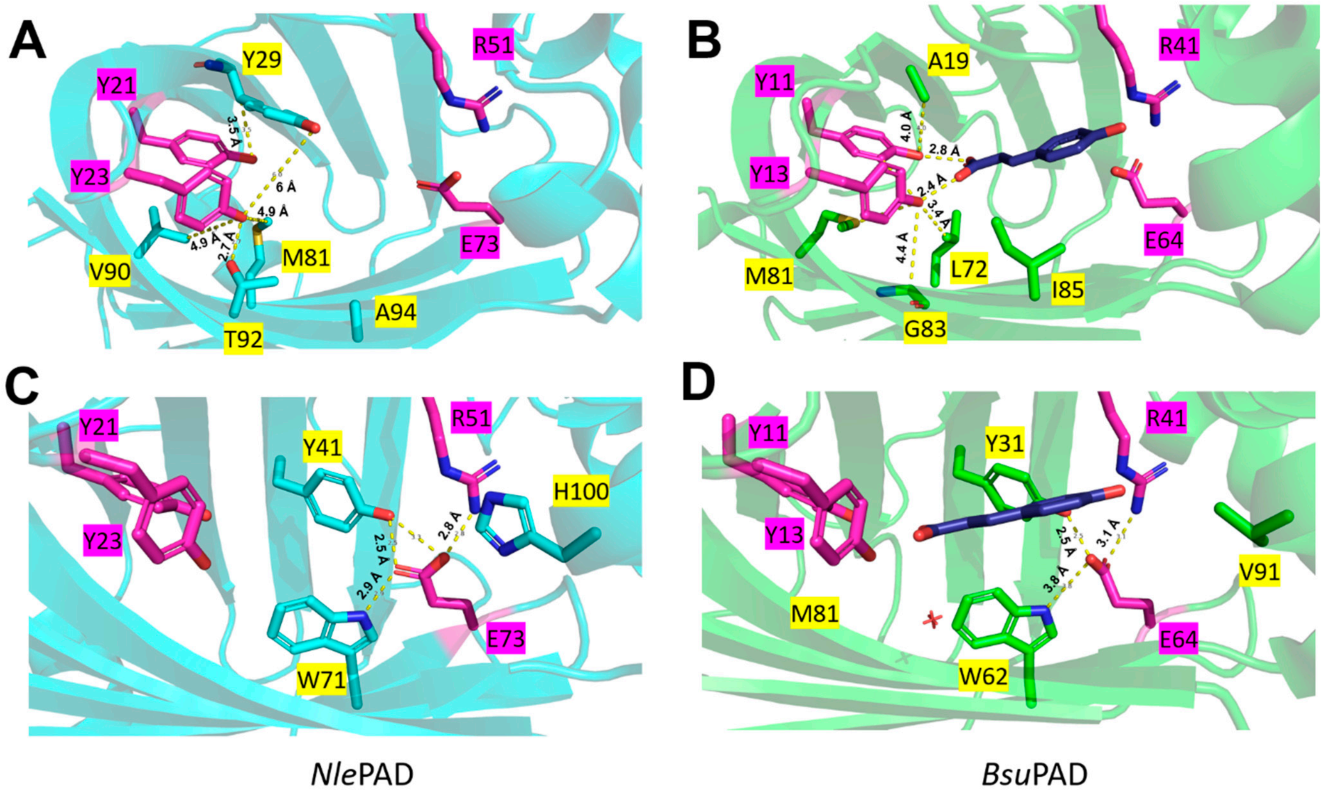

Figure S8. Neighborhood analysis of substrate-binding tyrosines and catalytic residues of PADs.

The figure shows the neighbor residues of **(A, B)** the substrate binding tyrosines (shown as magenta sticks), and **(C, D)** the catalytic residues Arg and Glu (shown as magenta sticks) for **(A, C)** *Nle*PAD (shown as blue carton; AlphaFold2 model; Jumper et al. 2021) and **(B, D)** *Bsu*PAD (shown as green cartoon; PDB code 4ALB; Frank et al. 2012). The structure of *Bsu*PAD (panels B and D) is that of *Bsu*PAD-Y19A mutant in complex with *p*-coumaric acid (shown as dark purple stick) (Frank et al. 2012).
